# Anxiety, depression and distress in family members of people who have experienced a critical care admission: a systematic review and Bayesian meta-analysis

**DOI:** 10.1186/s40560-025-00839-2

**Published:** 2025-12-15

**Authors:** P. Hartley, C. Brown, V. Danesh, F. Forsyth, K. Bond, I. Kuhn, M. Shaw, J. McPeake

**Affiliations:** 1 The Healthcare Improvement Studies Institute, University of Cambridge, Cambridge, UK; 2 Department of Physiotherapy, Cambridge University Hospitals NHS Foundation Trust, Cambridge, UK; 3 Center for Applied Health Research, Baylor Scott & White Health, Dallas, TX USA; 4 School of Medicine, Baylor College of Medicine, Temple, TX USA; 5 PACE Section (Perioperative, Acute, Critical Care and Emergency Medicine), Department of Medicine, University of Cambridge, Cambridge, UK; 6 KU Leuven Department of Public Health and Primary Care, Leuven, Belgium; 7 Medical Library, University of Cambridge, Cambridge, UK; 8 School of Medicine, Dentistry and Nursing, University of Glasgow, Glasgow, UK; 9 Clinical Physics, NHS Greater Glasgow and Clyde, Glasgow, UK

**Keywords:** Anxiety, Bayesian, Critical care, Depression, Family, Mental health, Meta-analysis, Systematic review

## Abstract

**Background:**

Family members of adults who experience a critical care admission often experience significant strain and emotional distress following discharge. This meta-analysis aimed to synthesise the levels of distress, anxiety, and depression in family members of people who have experienced a critical care admission.

**Methods:**

Medline, PsycINFO, Scopus, CINAHL and Web of Science databases were searched for articles (2000–2024) that measured distress using the Impact of Events Scale—Revised (IES-R), or anxiety or depression using the Hospital Anxiety and Depression Scale subscales (HADS-A and HADS-D) 3 months after critical care. Bayesian meta-analyses estimated the pooled average, and meta-regression examined whether the inclusion of bereaved relatives influenced the pooled outcome estimates. Anxiety and depression models estimated the pooled proportion of participants with HADS scores >7.

**Results:**

Fifty articles were included (45 cohorts). Seventeen studies were from the USA and the median sample size at baseline assessment was 94.0. The pooled estimate of the IES-R at 3 months was 18.16 points [95% credible interval (CrI): 12.26–26.23, 15 studies]. The pooled estimate of the HADS-A at 3 months was 5.98 points (CrI: 5.29–6.73, 35 studies). The estimated proportion of family members with clinically meaningful levels of anxiety (HADS-A > 7) was 0.38 (95% CrI: 0.30–0.47; 11 studies). The pooled estimate of the HADS-D at 3 months was 3.91 points (95% CrI: 3.39–4.50; 33 studies). The estimated proportion of family members with clinically meaningful levels of depression (HADS-D > 7) was 0.20 (95% CrI: 0.15–0.26; 11 studies). Meta-regression found no significant effect of including non-bereaved participants only with the HADS subscales and was not possible due to insufficient studies with the IES-R.

**Conclusions:**

Levels of distress, anxiety and depression appear to be comparable between individuals who experience a critical care admission and their family members. An estimated 38% and 20% of family members have clinically important levels of anxiety and depression, respectively.

*PROSPERO registration*: CRD42022302735.

**Supplementary Information:**

The online version contains supplementary material available at 10.1186/s40560-025-00839-2.

## Background

The number of patients surviving an admission to critical care is increasing [1]. As a result, there is greater recognition of the challenges faced by those who have experienced a critical care admission [2]. Following discharge, many experience ongoing physical, emotional, cognitive and social problems [3]. Consequently, many family members adopt the role of informal caregiver [4].

Evidence demonstrates that these family members also experience significant strain and emotional distress following hospital discharge for critical illness [5, 6]. For example, multi-centre data from Canada found that over two-thirds of informal caregivers experienced symptoms of depression in the months following critical care [7]. Similar findings have been reported in the UK, with up to 70% reporting symptoms of anxiety, and 40% experiencing financial strain [8]. Greater caregiver strain in informal caregivers is associated with an increased risk of hospital readmission for the person recovering from the critical care admission [9]. Despite descriptive research examining the outcomes of informal caregivers, there is limited high quality interventional research examining how their outcomes can be improved. Interventions including online resources and telephone support have been evaluated; but none have shown benefit in a randomised control trial (RCT) [10].

Given the pressing need to support family members, a comprehensive understanding of their experiences following a critical care admission is needed. With a substantial body of literature now available [11], this represents a timely opportunity to synthesise the evidence to inform clinical practice and to identify priorities for research, including the development of targeted interventions. Therefore, we conducted a systematic review and meta-analysis which synthesised levels of anxiety, depression, and distress in family members of people who have experienced a critical care admission. To inform clinical care planning and interventional research in this area, we used Bayesian methods to estimate posterior probabilities of the incidence of anxiety, depression and distress in family members.

## Methods

Ethical review was not required as this systematic review and meta-analysis only involved reviewing existing literature.

### Search strategy

Medline, PsycINFO, Scopus, CINAHL and Web of Science (Core Collection) databases were searched for publications between 2000 and 2024. We chose a start date of 2000 to reflect contemporary research and practice. The full search strategy is provided in the online supplementary materials (e-Tables 1–6), and was developed with an experienced medical librarian.

### Eligibility

We included published studies of any design that assessed distress, anxiety, or depression in family members of critically ill patients. Studies used multiple descriptions of informal caregivers, family members and loved ones. For this review, we have included all under the umbrella term of family members. Family members of patients who received care in any critical care setting, including general, neurological, or cardiac critical care units were included.

We excluded studies focused on bereavement of family members of people who died during critical care admission or before the follow-up assessment, but included mixed samples of family members of people who were alive at follow-up and of people who had died during follow-up. We excluded studies, where all participants received an intervention to influence anxiety, distress or depression in family members and retained data from control arms when applicable. Studies published only as abstracts, conference proceedings, theses, or non-English language studies, and those focused on neonatal and paediatric populations were excluded. We also excluded case reports, reviews, letters, and editorials.

### Outcomes

Studies that reported the Impact of Events Scale—Revised (IES-R) or the anxiety or depression subscales of the Hospital Anxiety or Depression Scale (HADS) were included. These were chosen as they were the most frequent measures used in studies of family members of adults admitted to critical care [11]. The decision to exclude other measures of distress, anxiety, or depression was made to reduce conceptual and statistical heterogeneity that could potentially be introduced by multiple measures with different properties and which do not necessarily measure the same construct. Given our a priori knowledge of the size of the available data [11], this decision was felt to be justified from a pragmatic stance as striking a balance between the comprehensiveness of the review, and a desire to maximise the coherence through attempting to reduce heterogeneity and optimising comparability and interpretation.

The IES-R consists of 22 items measuring the degree of distress an individual experiences in response to a traumatic event, with 8 items measuring intrusion, 8 items measuring avoidance, and 6 measuring hyperarousal [12]. Each item is rated on a five-point Likert scale from 0 (“not at all”) to 4 (“extremely”), giving a total score between 0 and 88, with higher scores indicating greater symptom severity [12].

The HADS consists of 14 items assessing mood, with 7 items measuring anxiety (HADS-A) and 7 measuring depression (HADS-D) [13]. Each subscale ranges from 0 to 21, with higher scores indicating greater levels of symptom severity. Scores of >7 on either subscale are considered indicative of clinically meaningful symptoms of anxiety or depression, respectively [13].

For the IES-R we excluded studies that only reported the proportion of individuals above or below a threshold on the scale. Although it is an endorsed tool to use in this population [2, 14], the clinical interpretation of a score above or below a threshold is questionable as it does not capture symptom domains necessary for a diagnosis of PTSD according to DSM-5 criteria [15, 16]. For the HADS outcomes we included studies that reported sample averages or proportion of study participants above or below 7 on the subscales (HADS-A or HADS-D).

We only included studies that reported the outcomes at 3 ± 1 months after critical care. As studies’ definitions of follow-up periods and admission durations varied, the timepoints could be 3 ± 1 months from any point between critical care admission and critical care discharge. The follow-up window of 3 ± 1 months was chosen as this was the most frequent assessment timepoint in the scoping review by Brown et al. [11] of all outcomes of family members of critically ill patients.

### Selection of studies

Two reviewers independently examined all titles and abstracts using predefined eligibility criteria. If a reason for exclusion from reading the title or abstract was not evident the article proceeded to full-text screening and the full manuscript was obtained. Full-text screening was also undertaken independently by two reviewers, with disagreements resolved by a third author.

### Data extraction

For interventional studies, only control group data were extracted. Relevant data were extracted by one reviewer and checked by a second reviewer. Data extracted included, study design, country, critical care unit type or patient condition, sample size, outcome measures, and follow-up assessment timepoints.

### Risk-of-bias assessments

Risk of bias was assessed with the quantitative section of the mixed-methods appraisal tool [17]. While this tool was originally designed for mixed-methods reviews, the tool is designed to be used to appraise the quality of purely quantitative studies (as well as other non-mixed-methods designs) [17]. The tool was felt to be proportionate and in line with the scope of this review. All assessments were checked by a second reviewer.

### Data synthesis

Analysis was conducted using R (version 4.3.2) [18]. Certain missing data were imputed using the following approaches. Where studies reported only medians, we used these as estimates of the mean. Interquartile ranges (IQRs) were converted to estimates of the standard deviations (SDs) by dividing the IQR by 1.35, and SDs were derived from related statistics when necessary, using established methods [19].

Where the IES-R was presented as the mean score per item (with SD), the overall mean was calculated by multiplying by 22 (items in IES-R). SDs were not derived from the domain-level SDs due to concerns of inter-domain correlation leading to underestimating the true variation. Instead, pooled SDs from studies reporting total IES-R scores were used.

All synthesis was undertaken using Bayesian random-effects models using the R brms package [20]. The planned analysis included three distinct models. The primary model estimated the weighted average of each outcome (distress, anxiety, and depression). The second model employed meta-regression to examine whether the inclusion of bereaved relatives influenced the pooled outcome estimates. The final model (anxiety and depression only) estimated the pooled proportion of participants with HADS scores >7. Model specifications are described in e-Table 7.

We calculated credible intervals (CrIs) and prediction intervals (PrIs) to describe the models. CrIs describe the uncertainty in the estimate of the pooled average (or proportion) given the data and priors. They can be interpreted as the range within which there is a 95% probability that the true pooled average (or proportion) lies. PrIs describe the expected range of estimates (averages or proportions) that might be observed in future studies, given the parameter uncertainty and between-study heterogeneity. Larger between-study heterogeneity will result in wider PrIs.

The primary analyses estimated the weighted averages and 95% CrI for each of the three outcome measures at 3 ± 1 months following critical care. A log-normal model was used to account for potential positive skew and the bounded nature of all three outcomes, as previous research of both family members and people who have experienced critical care admissions have estimated averages closer to 0 than the maximum values on the scales [21–24]. To inform future research and clinical practice, for both HADS anxiety and depression, the probability of a future study in this population reporting clinically relevant average scores (HADS > 7) was estimated.

Meta-regression models assessing the impact of including bereaved relatives were restricted to studies that reported whether bereaved relatives were included in the 3-month follow-up assessment. Meta-regression was only performed when the model included at least 10 studies, and when there were at least 3 studies in each group (i.e., those that did and did not include bereaved participants) [25]. These models included a binary covariate indicating whether bereaved family members were included (yes/no) to examine its association with outcome estimates. Apart from this addition, model specifications and priors matched those in the primary analyses.

The final models estimated the pooled proportion of participants with clinically relevant HADS scores (HADS > 7) [13]. Proportions from individual studies were converted to log-odds of proportions using the R metafor package [26], and synthesised in a Bayesian meta-analysis.

### Sensitivity analyses

Sensitivity analyses assessed the robustness of the primary analyses (weighted averages) and the HADS threshold models to prior specification by specifying more conservative priors for the pooled average (see e-Table 7).

Further sensitivity analyses assessed the impact of removing (the control arms) or randomised controlled trials, and separate sensitivity analyses assessed the impact of removing studies with higher than 20% non-response at 3-month follow-up.

All results were back transformed to aid interpretation. We assessed convergence using the potential scale reduction statistic ($\hat{R}$) and effective sample size (ESS).

## Results

**Fig. 1:**
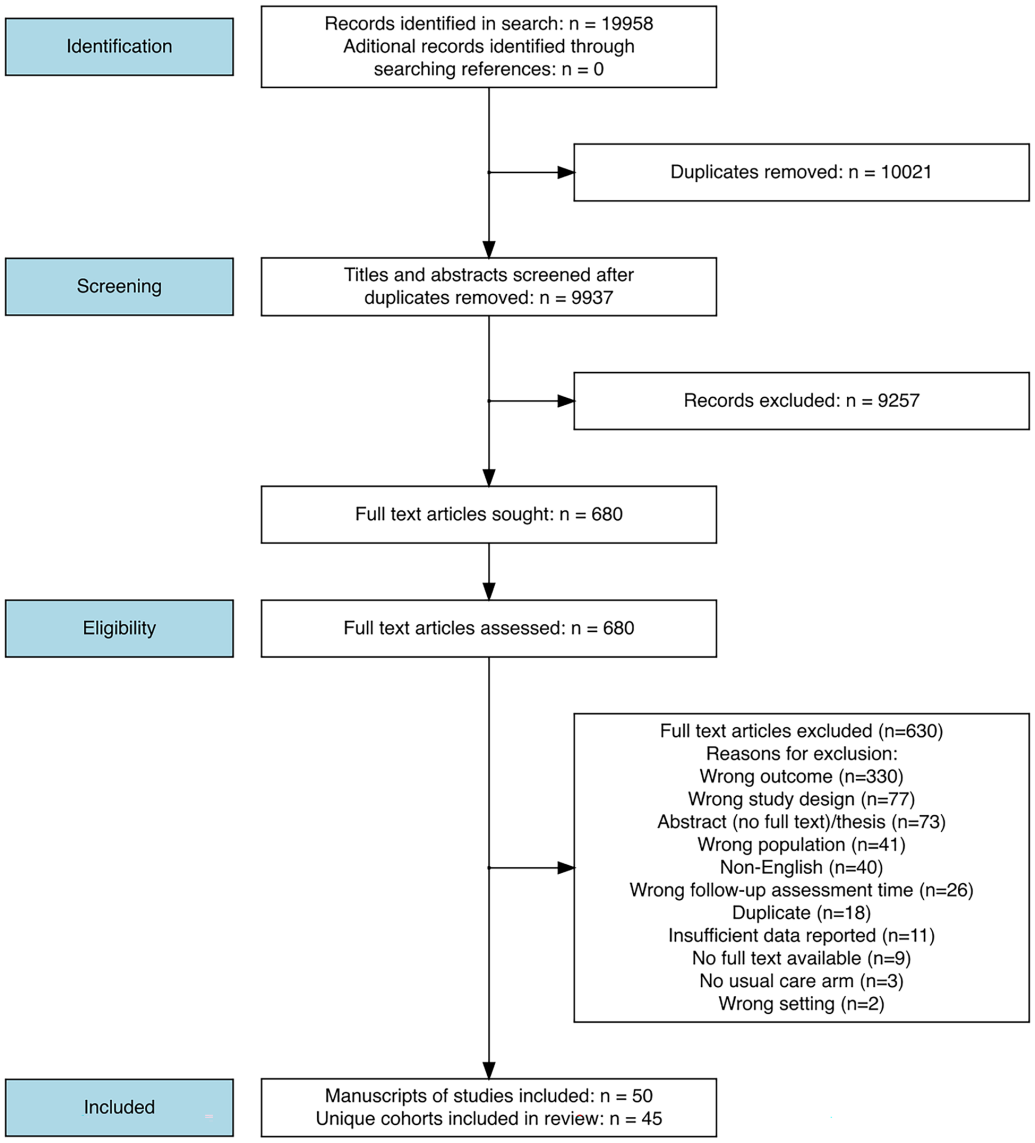
Study flow diagram

Our search returned 9937 unique articles, of which 50 articles met the inclusion criteria (Fig. 1). Following the identification of duplicate cohorts, 45 unique family member cohorts were included in this analysis. Study characteristics are presented in Table 1 [10, 27–74, 78], all 50 studies are referenced (in later figures, the study ID refers to the first instance, where the data plotted were reported). Studies were conducted in 15 countries, with 16 cohorts from the USA.

**Table 1:** Description of included studies

Study ID(s)	Country	Design	Population type	N careers at baseline	Includes bereaved careers	Outcomes relevant to this review
Amass et al. [27]	USA and Italy	Non-randomised experimental study	Mixed ICU (general/surgical)	226	Yes	HADS-A and HADS-D
Amass et al. [28]	Norway	Cohort/cross-sectional study	COVID-19	330	Yes	HADS-A and HADS-D
Azoulay et al. [29]	France and Belgium	Cohort/cross-sectional study	Mixed ICU (general/surgical)	421	Yes	HADS-A and HADS-D
Azoulay et al. [30]	France	Cohort/cross-sectional study	ARDS	602	Yes	IES-R, HADS-A and HADS-D
Bannon et al. [31]	USA	Randomised controlled trial	Neuro (surgical/trauma)	10	Not stated	HADS-A and HADS-D
Beesley et al. [32, 33] and Harris et al. [34]	USA	Cohort/cross-sectional study	Mixed ICU (general/surgical)	99	Yes	HADS-A and HADS-D
Bohart et al. [35]	Denmark	Randomised controlled trial	Mixed ICU (general/surgical)	94	No	HADS-A and HADS-D
Carson et al. [36]	USA	Randomised controlled trial	Mixed ICU (general/surgical)	181	Yes	IES-R, HADS-A and HADS-D
Cattelan et al. [37]	France	Cohort/cross-sectional study	COVID-19	88	Yes	IES-R
Cox et al. [10, 38]	USA	Randomised controlled trial	Mixed ICU (general/surgical)	47	Yes	IES-R, HADS-A and HADS-D
de Miranda et al. [39]	France	Cohort/cross-sectional study	Other: COPD	102	Not stated	IES-R, HADS-A and HADS-D
de Ridder et al. [40]	Netherlands	Cohort/cross-sectional study	Mixed ICU (general/surgical)	86	Yes	IES-R, HADS-A and HADS-D
Dijkstra et al. [41]	Netherlands	Randomised controlled trial	Mixed ICU (general/surgical)	233	Not stated	IES-R^a^, HADS-A and HADS-D
Fumis et al. [42]	Brazil	Cohort/cross-sectional study	Mixed ICU (general/surgical)	184	Yes	HADS-A and HADS-D
Fumis et al. [43]	Brazil	Cohort/cross-sectional study	Mixed ICU (general/surgical)	186	Yes	HADS-A and HADS-D
Garrouste-Orgeas et al. [44]	France	Cohort/cross-sectional study	Mixed ICU (general/surgical)	48	Yes	HADS-A and HADS-D
Garrouste-Orgeas et al. [45]	France	Randomised controlled trial	Mixed ICU (general/surgical)	50	Yes	IES-R, HADS-A and HADS-D
Garrouste-Orgeas et al. [46]	France	Randomised controlled trial	Mixed ICU (general/surgical)	325	Yes	IES-R, HADS-A and HADS-D
Gonzalez-Martin et al. [47]	Spain	Randomised controlled trial	Cardio-thoracic critical care	19	No	IES-R, HADS-A and HADS-D
Greenleaf et al. [48]	USA	Cohort/cross-sectional study	Mixed ICU (general/surgical)	45	Yes	IES-R
Harlan et al. [49]	USA	Other: mixed methods	Mixed ICU (general/surgical)	40	Yes	HADS-A and HADS-D
Heesakkers et al. [50]	Netherlands	Cohort/cross-sectional study	COVID-19	197	No	HADS-A and HADS-D
Henderson et al. [51]	Scotland	Quality improvement	Cardio-thoracic critical care	20	No	HADS-A and HADS-D
Hickman et al. [52]	USA	Cohort/cross-sectional study	Mixed ICU (general/surgical); Neuro (surgical/trauma)	101	Not stated	HADS-A and HADS-D
Kentish-Barnes et al. [53]	France	Randomised controlled trial	Mixed ICU (general/surgical)	189	No	HADS-A and HADS-D
Komachi and Kamibeppu [54]	Japan	Cohort/cross-sectional study	Mixed ICU (general/surgical)	26	Not stated	IES-R
Lester et al. [55]	USA	Cohort/cross-sectional study	Neuro (surgical/trauma)	96	Yes	HADS-A
Lobato et al. [78]	Portugal	Cohort/cross-sectional study	Mixed ICU (general/surgical)	246	No	HADS-A and HADS-D
Matt et al. [56]	Germany	Cohort/cross-sectional study	Sepsis	143	Only extracted data of non-bereaved	HADS-A and HADS-D
McAdam et al. [57]	USA	Cohort/cross-sectional study	Mixed ICU (general/surgical)	41	Yes	IES-R^a^, HADS-A and HADS-D
Meyers et al. [58–60]	USA	Cohort/cross-sectional study	Neuro (surgical/trauma)	103	Not stated	HADS-A and HADS-D
Milton et al. [61]	Sweden	Cohort/cross-sectional study	Mixed ICU (general/surgical)	62	No	HADS-A and HADS-D
Oliveira and Fumis [62]	Brazil	Cohort/cross-sectional study	Mixed ICU (general/surgical)	118	Yes	HADS-A and HADS-D
Petrinec et al. [63]	USA	Cohort/cross-sectional study	Mixed ICU (general/surgical)	112	Yes	IES-R
Petrinec [64]	USA	Cohort/cross-sectional study	Mixed ICU (general/surgical)	30	Yes	HADS-A and HADS-D
Petrinec and Martin [65]	USA	Cohort/cross-sectional study	Mixed ICU (general/surgical)	48	Yes	HADS-A and HADS-D
Petrinec et al. [66]	USA	Randomised controlled trial	Mixed ICU (general/surgical)	25	Yes	HADS-A and HADS-D
Petrinec et al. [67]	USA	Randomised controlled trial	Mixed ICU (general/surgical)	30	Yes	HADS-A and HADS-D
Torres et al. [68]	Portugal	Cohort/cross-sectional study	Mixed ICU (general/surgical)	168	No	HADS-A and HADS-D
van Veenendaal et al. [69]	Netherlands	Cohort/cross-sectional study	COVID-19	102	No	HADS-A and HADS-D
Veislinger-Burelli et al. [70]	France	Non-randomised experimental study	Mixed ICU (general/surgical)	77	Not stated	HADS-A and HADS-D
Viana et al. [71]	Brazil	Cohort/cross-sectional study	Mixed ICU (general/surgical)	73	Only extracted data of non-bereaved	HADS-A and HADS-D
Vranceanu et al. [72]	USA	Randomised controlled trial	Neuro (surgical/trauma)	29	Not stated	HADS-A and HADS-D
Wiertz et al. [73]	Netherlands	Cohort/cross-sectional study	COVID-19	57	Not stated	HADS-A and HADS-D
Zante et al. [74]	Switzerland	Cohort/cross-sectional study	Mixed ICU (general/surgical)	72	Yes	IES-R

*ARDS* acute respiratory distress syndrome, *IES-R* the Impact of Event Scale—Revised, *HADS-A* Hospital Anxiety and Depression Scale—Anxiety, *HADS-D* Hospital Anxiety and Depression—Depression

^a^IES-R was presented as the mean score per domain, calculation of overall mean and imputation of SD described in “Methods” section

Twelve were randomised controlled studies, from which control arm data were extracted. The median sample size at baseline assessment was 94.0 (IQR: 47.0–181.0). Five studies were specific to COVID-19-related critical illness, and four included patients with COVID-19 as part of their cohort.

### Risk of bias

The risk of bias assessment highlighted risk of non-response bias in many studies (see e-Tables 8–10). We defined risk of non-response bias as having data for <80% of the recruited participants at the 3 ± 1-month assessments. Risk of non-response bias was identified in 16 (40.0%) studies measuring HADS-A, 16 (41.0%) studies measuring HADS-D and 5 (33.3%) studies measuring IES-R. Median follow-up rates at 3 ± 1 months were 86.1% for IES-R, 83.9% for HADS-A, and 83.5% for HADS-D.

### Overview of Bayesian approach

All models showed good convergence (all potential scale reduction statistics ($\hat{R}$) = 1.00; effective sample size (ESS) > 5000), e-Table 11 provides further details of Markov chain Monte Carlo (MCMC) chain convergence and resolution. Posterior predictive checks are presented in e-Figure A.

### IES-R

**Fig. 2:**
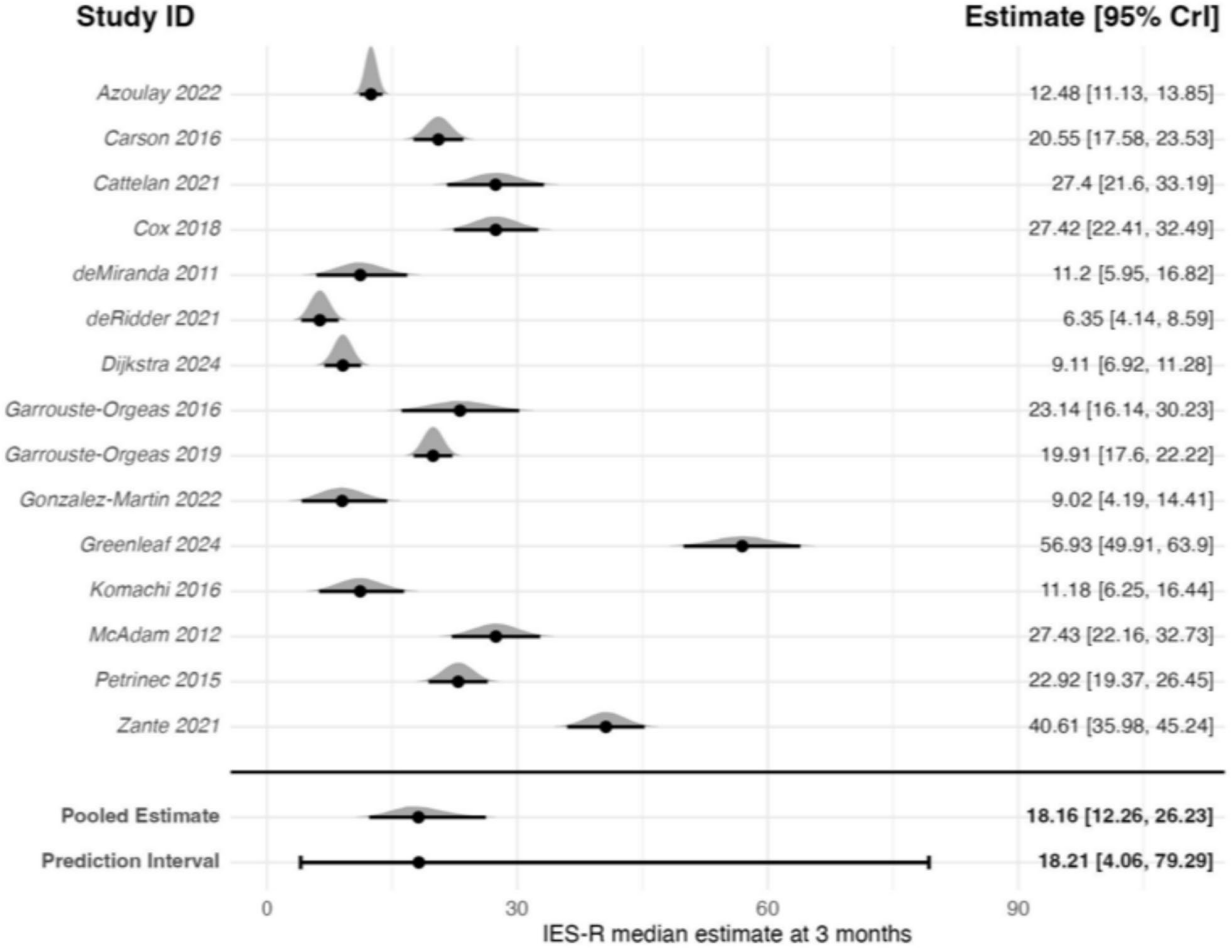
Forest plot of IES-R estimates for family members 3 ± 1-month post-critical care

The primary analysis (weighted average) meta-analysis of 15 studies (1640 participants) estimated a back transformed pooled estimate of 18.16 (95% CrI: 12.26–26.23) on the IES-R scale (0–88) (Fig. 2). Between-study heterogeneity was high (*τ* = 1.97), with a pooled 95% PrI of 4.06–79.29 points, indicating substantial variability in expected study-level effects.

Sensitivity analysis with a prior assuming a mean of 0, did not meaningfully change the results (pooled estimate: 18.02; 95% CrI: 12.19–26.16). Only one study included only non-bereaved relatives at 3-month follow-up; therefore, no meta-regression analysis was conducted. In further sensitivity analyses, neither removing the RCTs, or studies with high non-response (>20%) meaningfully changed the results as indicated by the inclusive CrIs and high heterogeneity. The non-RCTs sensitivity analysis of 9 studies (878 participants) estimated a back transformed pooled estimate of 19.24 (95% CrI: 10.14–36.05; *τ* = 2.29). The sensitivity analysis removing studies with high non-response of 10 studies (1447 participants) estimated a back transformed pooled estimate of 16.77, (95% CrI: 10.08–27.12; *τ* = 2.29).

### HADS-anxiety

**Fig. 3:**
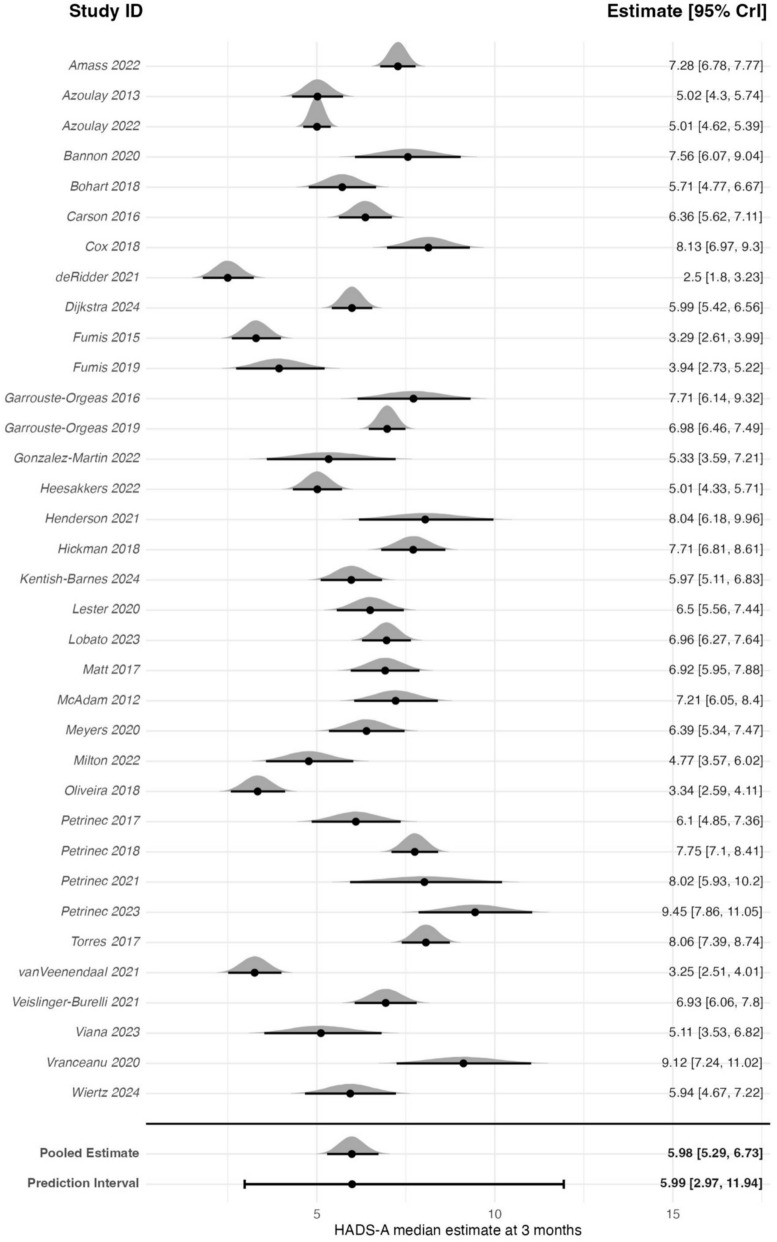
Forest plot of HADS-A estimates for family members 3 ± 1-month post-critical care

The meta-analysis of 35 studies (3606 participants) estimated a back transformed pooled estimate of 5.98 (95% CrI: 5.29–6.73) on the HADS-A scale (0–21) (Fig. 3). Between-study heterogeneity was high (*τ* = 1.47) resulting in a pooled 95% PrI of 2.97–11.94. The estimated probability that a future study will report an average HADS-A score exceeding the clinically meaningful threshold of seven was 37%.

Sensitivity analysis with a prior assuming a mean of 0, did not meaningfully change the results (pooled estimate: 5.98; 95% CrI: 5.28–6.74). A sub-analysis was conducted of studies reporting the inclusion of bereaved family members at the 3-month follow-up assessment. In the meta-regression of 28 studies (3036 participants), 11 studies (976 participants) only included family members of survivors. The model included a binary covariate indicating whether bereaved family members were included (yes/no). The model estimated a pooled estimate of 5.73 (95% CrI: 4.72–6.92) on the HADS-A scale. Including non-bereaved participants only was associated with a 1% lower HADS-A score (coefficient estimate on the log scale: −0.01; 95% CrI: −0.32 to 0.30), suggesting similar levels of anxiety in studies that did and did not include bereaved relatives. By calculating Bayesian *R*^2^ it was estimated that the survival variable explained 0.00% of the observed between-study heterogeneity. In further sensitivity analyses, neither removing the RCTs, or studies with high non-response (>20%) meaningfully changed the results. The non-RCTs sensitivity analysis of 23 studies (2544 participants) estimated a back transformed pooled estimate of 5.43 (95% CrI: 4.56–6.42; *τ* = 1.46). The sensitivity analysis removing studies with high non-response of 22 studies (2430 participants) estimated a back transformed pooled estimate of 6.59 (95% CrI: 5.74–7.56; *τ* = 1.35).

**Fig. 4:**
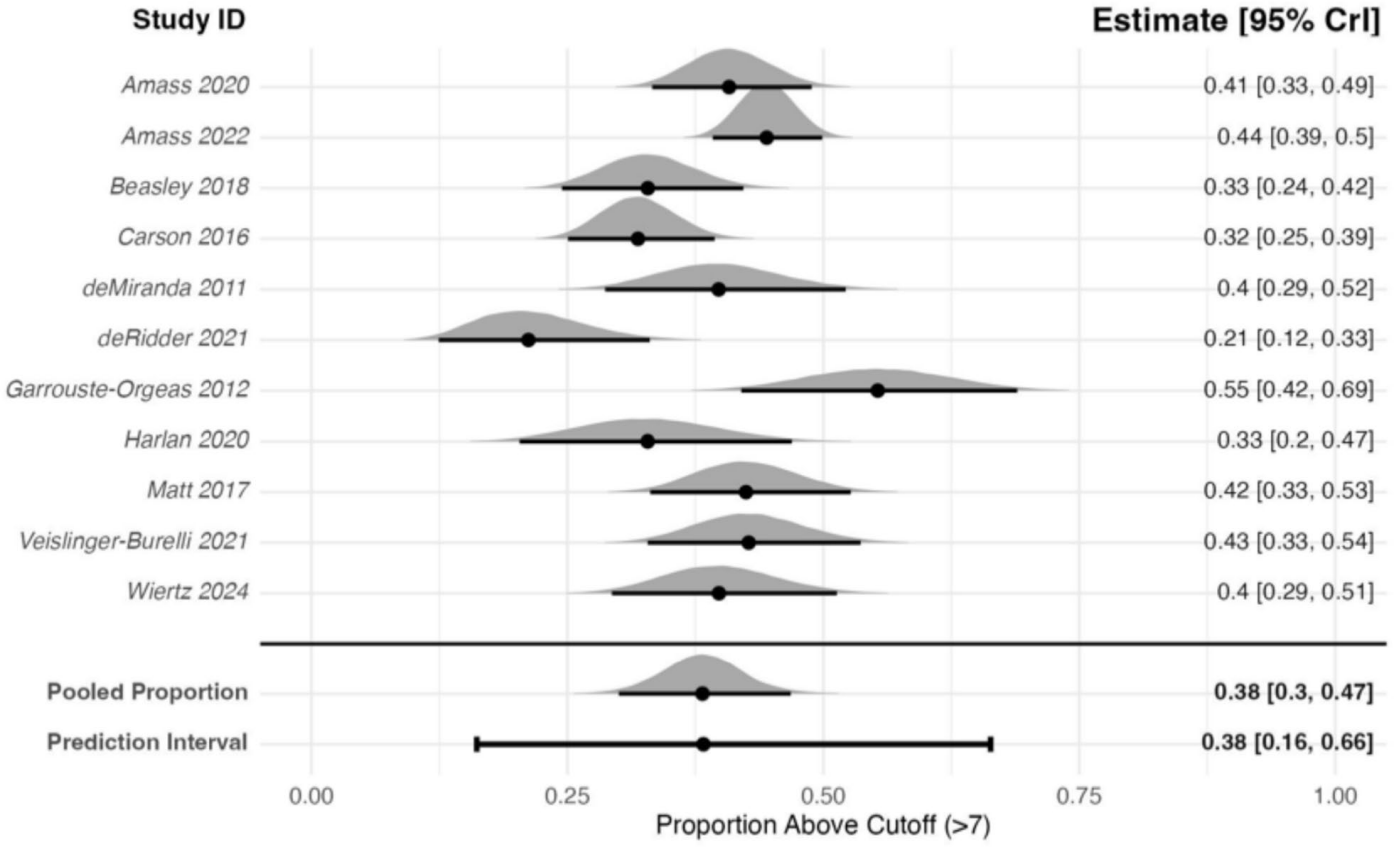
Forest plot of family members with HADS-A scores ≥8 at 3 ± 1-month post-critical care

The meta-analysis of 11 studies (1066 participants) that reported proportions of participants with HADS-A scores of more than 7 at 3 months after critical care admission estimated a pooled proportion of 0.38 (95% CrI: 0.30–0.47) (Fig. 4). Between-study heterogeneity was high (*τ* = 0.54), resulting in a pooled 95% PrI of 0.16–0.66. A sensitivity analysis using a prior centred on a mean proportion of 0, did not meaningfully change the results (pooled estimate: 0.38; 95% CrI: 0.29–0.46), indicating that the results were robust to prior specification. In further sensitivity analyses, neither removing the RCTs, or studies with high non-response (>20%) meaningfully changed the results. The non-RCTs sensitivity analysis of 9 studies (852 participants) estimated a pooled proportion of 0.38 (95% CrI: 0.29–0.46; *τ* = 0.58). The sensitivity analysis removing studies with high non-response of 7 studies (806 participants) estimated a pooled proportion of 0.33, (95% CrI: 0.24–0.54; *τ* = 0.70).

### HADS-depression

**Fig. 5:**
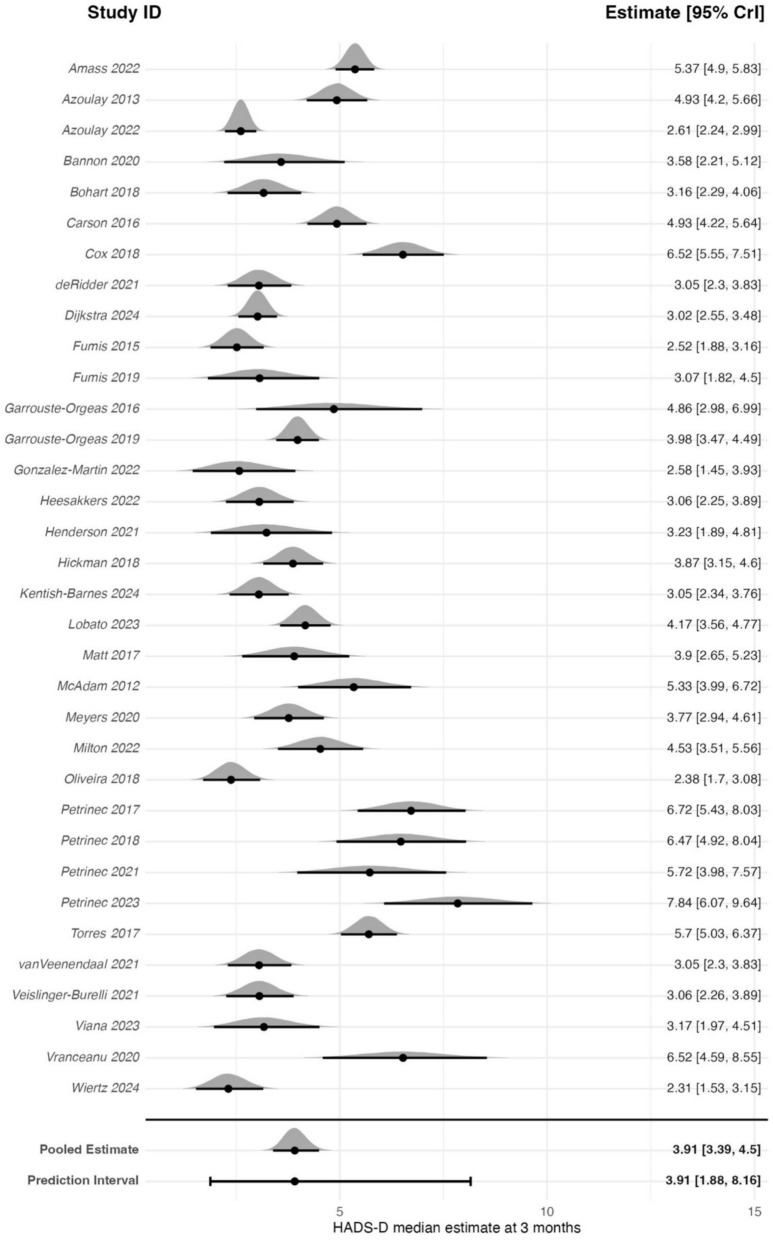
Forest plot of HADS-D estimates for family members at 3 ± 1-month post-critical care

The meta-analysis of 34 studies (3522 participants) estimated a back transformed pooled estimate of 3.91 (95% CrI: 3.39–4.50) on the HADS-D scale (0–21) (Fig. 5). Between-study heterogeneity was high (*τ* = 1.46) resulting in a pooled 95% PrI of 1.88–8.16. The estimated probability that a future study will report an average HADS-D score exceeding the clinically meaningful threshold of seven was 6.97%.

Sensitivity analysis with a prior assuming a mean of 0, did not meaningfully change the results (pooled estimate: 3.91; 95% CrI: 3.38–4.48). A sub-analysis was conducted of studies that reported whether they included bereaved family members at the 3-month follow-up assessment. In the meta-regression of 26 studies (2800 participants), 8 studies (635 participants) only included family members of survivors. The model included a binary covariate indicating whether bereaved family members were included (yes/no). The model estimated a pooled estimate of 4.21 (95% CrI: 3.42–5.15) on the HADS-D scale. Including non-bereaved participants only was associated with a 23% lower HADS-D score (coefficient estimate on the log scale: −0.26; 95% CrI: −0.64 to 0.11), indicating no clear evidence of a difference associated with excluding bereaved family members. Bayesian *R*^2^ showed a negligible change (−0.71%) when including the survival variable, indicating it explained virtually none of the observed between-study heterogeneity. In further sensitivity analyses, neither removing the RCTs, or studies with high non-response (>20%) meaningfully changed the results. The non-RCTs sensitivity analysis of 22 studies (2460 participants) estimated a back transformed pooled estimate of 3.68 (95% CrI: 3.07–4.35; *τ* = 1.44). The sensitivity analysis removing studies with high non-response of 21 studies (2346 participants) estimated a back transformed pooled estimate of 4.26, (95% CrI: 3.49–5.15; *τ* = 1.51).

**Fig. 6:**
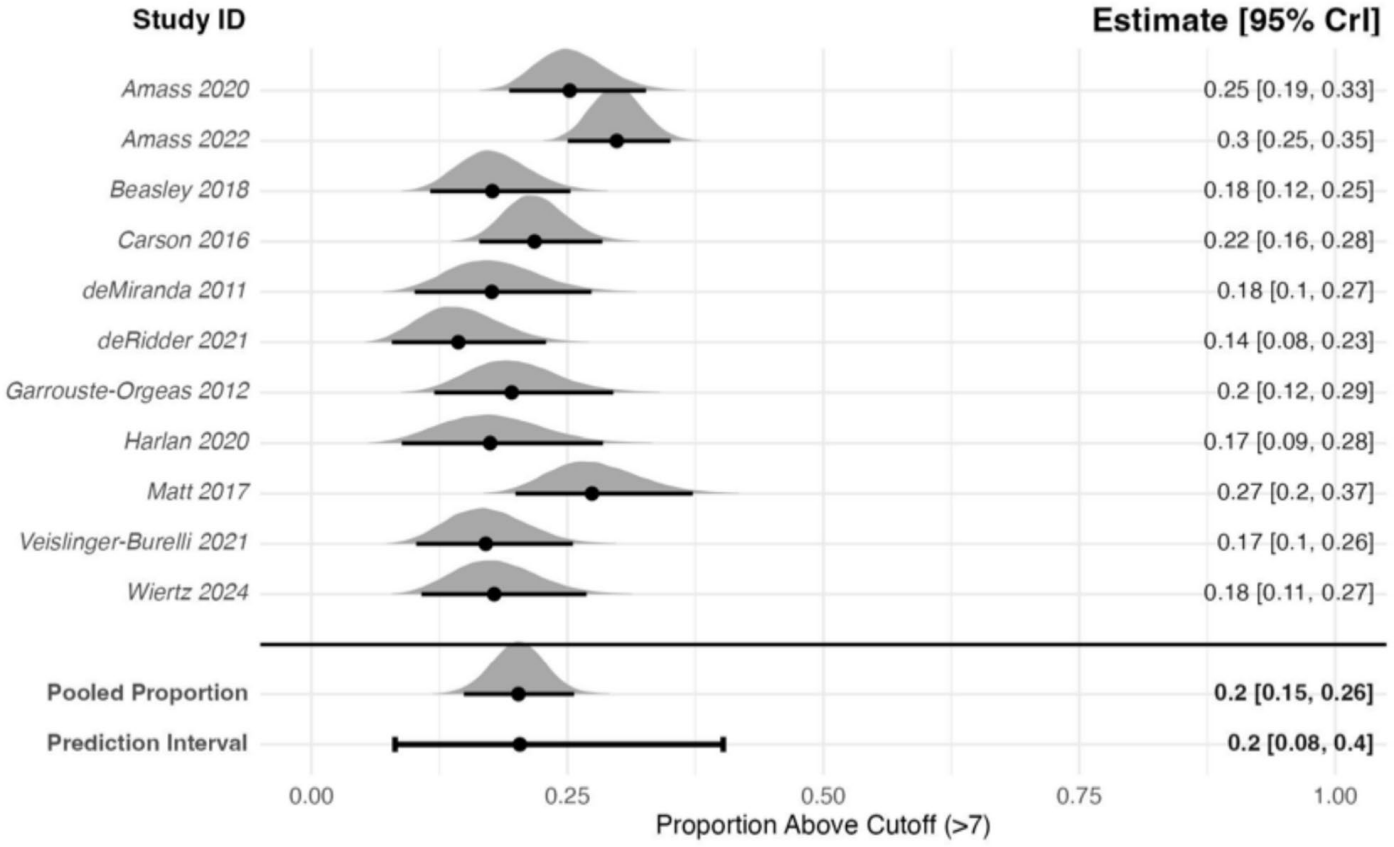
Forest plot of family members with HADS-D scores ≥8 at 3 ± 1-month post-critical care

The meta-analysis of 11 studies (1092 participants) that reported proportions of participants with HADS-D scores of >7 at 3 months after critical care admission estimated a pooled proportion of 0.20 (95% CrI: 0.15–0.26), see Fig. 6. Between-study heterogeneity was high (*τ* = 0.41), resulting in a pooled 95% PrI of 0.08–0.40. A sensitivity analysis using a prior centred on a mean proportion of 0, did not meaningfully change the results (pooled proportion: 0.20; 95% CrI: 0.15–0.25). In further sensitivity analyses, neither removing the RCTs, or studies with high non-response (>20%) meaningfully changed the results. The non-RCTs sensitivity analysis of 10 studies (943 participants) estimated a pooled proportion of 0.19 (95% CrI: 0.14–0.26; *τ* = 0.46). The sensitivity analysis removing studies with high non-response of 7 studies (805 participants) estimated a pooled proportion of 0.25, (95% CrI: 0.12–0.27; *τ* = 0.46).

## Discussion

This systematic review and Bayesian meta-analysis which included 45 unique family cohorts, shows that family members of people who have experienced a critical care admission have clinically important levels of distress, anxiety and depression in the months following exposure to critical illness. It provides estimates of these important outcomes to inform future research. Interestingly, it has demonstrated that those who have not experienced bereavement have similar rates of depression, anxiety and distress to those who have. Future research in this area is urgently needed to ensure that the entire family unit is supported following a critical care admission.

The findings relating to anxiety may be better contextualised by considering the proportion of family members with clinically relevant levels of anxiety. In this analysis, the estimated probability that a future study will report an average HADS-A score of >7 at 3 months, was 37%, and proportion of family members with anxiety was estimated at 38%. Previous patient cohorts have estimated anxiety prevalence to be 32%, 2–3 months following hospital discharge [24]. Although methods differ, these findings suggest that family members have similar rates of anxiety. While the mechanisms which drive anxiety in patients and family members are likely to differ, this evidence is important to optimise outcomes for people who have experienced a critical care admission. Future research should explicitly include family members in recovery programmes and consider the entire social unit during recovery.

The primary motivation for employing Bayesian methods in this review was to estimate the probability that future studies would report average HADS scores exceeding clinically meaningful thresholds. Unlike traditional frequentist approaches, Bayesian methods provide direct, interpretable probability estimates, which are particularly valuable for informing future research and developing targeted interventions.

Although the IES-R is recommended by expert consensus groups as an outcome measure for people who have a critical care admission [2, 14], it is a screening tool for acute stress disorder (<30 days of symptoms) and post-traumatic stress disorder (30 days or longer), and does not capture DSM-5 diagnostic criteria for PTSD. Its interpretation is further limited by various proposed cutoff thresholds [15, 16]. In this study, pooled IES-R scores of family members were comparable to those reported in a meta-analysis of patients 1–6 months after critical care (20; 95% CI 17–24; 6 studies, *I*^2^ = 78%) [21]. This suggests family members may experience similar levels of distress to the people who have had a critical care admission. These findings reinforce the importance of developing interventions to support family members of people who survive a critical care admission.

There is limited evidence to guide the implementation of interventions to support family members of survivors, though information provision and enhanced family communication have shown benefits for palliative and bereavement care [75]. Recent quality improvement research has shown that the inclusion of family members in ICU recovery programmes is feasible and may improve outcomes such as depression for family members [5]. Although combining interventions for family members and survivors is acceptable, evidence has also shown that trajectories of symptoms such as anxiety and depression for family members and patients differ over the 12 months after discharge [76]. Future research should examine both the effectiveness of interventions to support family members and the longitudinal pathway of these emotional issues to ensure sustained clinical impact.

While supporting family members (and caregivers) might seem outside the remit of critical care clinicians or the acute hospital system, it is important to recognise the potential impact of not supporting this cohort. Research has demonstrated patients whose family members are ‘strained’ are more likely to be readmitted within 12 months of discharge [9]. Supporting this vulnerable cohort is, therefore, likely to benefit patients, family members and the healthcare system.

### Limitations

This systematic review and meta-analysis has limitations. All analyses had high heterogeneity. We have included prediction intervals to support interpretation of our findings within the context of this heterogeneity. Prediction intervals should be interpreted as the expected range in which future studies may estimate the outcome [77]. As evident from the prediction intervals there is large uncertainty as to the likely findings of future studies. We hypothesised that some of the heterogeneity would be explained by the use of a dichotomous variable indicating whether studies included bereaved relatives. However, this variable accounted for little to no between-study variation. Had the included studies provided more consistent reporting on bereavement status, it may have been possible to construct a continuous variable representing the proportion of bereaved relatives per study, which might have explained more heterogeneity. Similarly, removing RCTs or studies with high non-response at 3-month follow-up (>20%) in sensitivity analyses did not provide meaningfully different interpretations of results. Variation in critical care unit types (e.g., general critical care units, and neuro-critical care units) may also contribute to heterogeneity. However, international differences in unit organisation, and because similar patient populations such as those with brain injury may be treated across different unit types, means restricting by unit type could mislead interpretations without addressing the source of the heterogeneity. We also restricted the outcome measures included in the review in an effort to reduce heterogeneity, this decision likely reduced the comprehensiveness of the review. Similarly, by limiting to English language publications we may further have reduced the comprehensiveness of the review. Finally, while we endeavoured to conduct a comprehensive search of the literature, it is possible that including extra databases such as Embase or the Cochrane Central Register of Controlled Trials we would have found additional papers to include.

## Conclusion

This systematic review and meta-analysis has demonstrated that many family members of people who have experienced a critical care admission have clinically important levels of anxiety and depression in the months following exposure to critical illness. The Bayesian methods offer direct interpretable probability estimates that future studies could report average scores exceeding clinically meaningful thresholds using the predominant screening tools for anxiety and depression (HADS). To ensure optimal outcomes, future research in this area is urgently needed to ensure that the entire family unit is supported following a critical care admission.

## Supplementary Information

Additional file 1.

## Data Availability

Data sharing is not applicable to this article as all primary data are presented in original manuscripts. Model specifications and search strategy are presented in online supplementary materials.

## Abbreviations

ARDS: Acute respiratory distress syndrome
CrI: Credible interval
ESS: Effective sample size
IES-R: The Impact of Event Scale—Revised
IQR: Interquartile range
HADS-A: Hospital Anxiety and Depression Scale—Anxiety
HADS-D: Hospital Anxiety and Depression—Depression
MCMC: Markov chain Monte Carlo
PrI: Prediction interval
SD: Standard deviation

## References

[1] Needham DM, Bronskill SE, Calinawan JR, Sibbald WJ, Pronovost PJ, Laupacis A. Projected incidence of mechanical ventilation in Ontario to 2026: preparing for the aging baby boomers. Crit Care Med. 2005;33(3):574–9. 15753749 10.1097/01.ccm.0000155992.21174.31

[2] Mikkelsen ME, Still M, Anderson BJ, et al. Society of Critical Care Medicine’s international consensus conference on prediction and identification of long-term impairments after critical illness. Crit Care Med. 2020;48(11):1670–9. 32947467 10.1097/CCM.0000000000004586

[3] Haines KJ, Hibbert E, McPeake J, et al. Prediction models for physical, cognitive, and mental health impairments after critical illness: a systematic review and critical appraisal. Crit Care Med. 2020;48(12):1871–80. 33060502 10.1097/CCM.0000000000004659 PMC7673641

[4] Sevin CM, Boehm LM, Hibbert E, et al. Optimizing critical illness recovery: perspectives and solutions from the caregivers of ICU survivors. Crit Care Explor. 2021;3(5):e0420. 34079948 10.1097/CCE.0000000000000420 PMC8162533

[5] McPeake J, Henderson P, MacTavish P, et al. A multicentre evaluation exploring the impact of an integrated health and social care intervention for the caregivers of ICU survivors. Crit Care. 2022;26(1):152. 35610616 10.1186/s13054-022-04014-z PMC9128318

[6] Haines KJ, Quasim T, McPeake J. Family and support networks following critical illness. Crit Care Clin. 2018;34(4):609–23. 30223998 10.1016/j.ccc.2018.06.008

[7] Cameron JI, Chu LM, Matte A, et al. One-year outcomes in caregivers of critically ill patients. N Engl J Med. 2016;374(19):1831–41. 27168433 10.1056/NEJMoa1511160

[8] McPeake J, Devine H, MacTavish P, et al. Caregiver strain following critical care discharge: an exploratory evaluation. J Crit Care. 2016;35:180–4. 27481756 10.1016/j.jcrc.2016.05.023

[9] Docherty C, Shaw M, Chim CY, MacTavish P, Devine H, O’Brien P, et al. Association between caregiver strain and emergency health care resource utilization in survivors of critical illness. Chest. 2025;167(3):768–71. 39368736 10.1016/j.chest.2024.08.057 PMC11882738

[10] Cox CE, Hough CL, Carson SS, et al. Effects of a telephone- and web-based coping skills training program compared with an education program for survivors of critical illness and their family members. A randomized clinical trial. Am J Respir Crit Care Med. 2018;197(1):66–78. 28872898 10.1164/rccm.201704-0720OC

[11] Brown C, Hartley P, Forsyth F, et al. What measures have been used to explore the outcomes of family members of critically ill patients: a scoping review. Intensive Care Med. 2025;51(9):1641–50. 40794169 10.1007/s00134-025-08072-z PMC12405393

[12] Weiss DS, Marmar CR. The impact of event scale—revised. In: Wilson JP, Keane TM, editors. Assessing psychological trauma and PTSD. New York: Guilford Press; 1997. p. 399–411.

[13] Zigmond AS, Snaith RP. The hospital anxiety and depression scale. Acta Psychiatr Scand. 1983;67(6):361–70. 6880820 10.1111/j.1600-0447.1983.tb09716.x

[14] Dinglas VD, Faraone LN, Needham DM. Understanding patient-important outcomes after critical illness: a synthesis of recent qualitative, empirical, and consensus-related studies. Curr Opin Crit Care. 2018;24(5):401–9. 30063492 10.1097/MCC.0000000000000533 PMC6133198

[15] Beck JG, Grant DM, Read JP, et al. The impact of event scale-revised: psychometric properties in a sample of motor vehicle accident survivors. J Anxiety Disord. 2008;22(2):187–98. 17369016 10.1016/j.janxdis.2007.02.007 PMC2259224

[16] Morina N, Ehring T, Priebe S. Diagnostic utility of the impact of event scale-revised in two samples of survivors of war. PLoS ONE. 2013;8(12):e83916. 24391844 10.1371/journal.pone.0083916 PMC3877127

[17] Hong QN, Fàbregues S, Bartlett G, et al. The mixed methods appraisal tool (MMAT) version 2018 for information professionals and researchers. Educ Inf. 2018;34(4):285–91.

[18] R: A language and environment for statistical computing. R Foundation for Statistical Computing [computer program], Vienna, Austria. 2025.

[19] Higgins JPT, Li T, Deeks JJ. Chapter 6: Choosing effect measures and computing estimates of effect. In: Higgins JPT, Thomas J, Chandler J, et al., editors. Cochrane handbook for systematic reviews of interventions (version 6.5.). Cochrane. 2024.

[20] Bürkner P-C. brms: an R package for Bayesian multilevel models using Stan. J Stat Softw. 2017;80(1):1–28.

[21] Parker AM, Sricharoenchai T, Raparla S, Schneck KW, Bienvenu OJ, Needham DM. Posttraumatic stress disorder in critical illness survivors: a metaanalysis. Crit Care Med. 2015;43(5):1121–9. 25654178 10.1097/CCM.0000000000000882

[22] White DB, Angus DC, Shields AM, et al. A randomized trial of a family-support intervention in intensive care units. N Engl J Med. 2018;378(25):2365–75. 29791247 10.1056/NEJMoa1802637

[23] Rabiee A, Nikayin S, Hashem MD, et al. Depressive symptoms after critical illness: a systematic review and meta-analysis. Crit Care Med. 2016;44(9):1744–53. 27153046 10.1097/CCM.0000000000001811 PMC7418220

[24] Nikayin S, Rabiee A, Hashem MD, et al. Anxiety symptoms in survivors of critical illness: a systematic review and meta-analysis. Gen Hosp Psychiatry. 2016;43:23–9. 27796253 10.1016/j.genhosppsych.2016.08.005 PMC5289740

[25] Deeks JJ, Higgins JPT, Altman DG, editors. Chapter 10: Analysing data and undertaking meta-analyses. In: Higgins JP, Thomas J, Chandler J, et al., editors. Cochrane handbook for systematic reviews of interventions (version 6.5.). Version 6.3 ed. Cochrane. 2024.

[26] Viechtbauer W. Conducting meta-analyses in R with the metafor package. J Stat Softw. 2010;36(3):1–48.

[27] Amass TH, Villa G, OMahony S, et al. Family care rituals in the ICU to reduce symptoms of post-traumatic stress disorder in family members—a multicenter, multinational, before-and-after intervention trial. Crit Care Med. 2020;48(2):176–84. 31939785 10.1097/CCM.0000000000004113 PMC7147959

[28] Amass T, Van Scoy LJ, Hua M, et al. Stress-related disorders of family members of patients admitted to the intensive care unit with COVID-19. JAMA Intern Med. 2022;182(6):624–33. 35467698 10.1001/jamainternmed.2022.1118 PMC9039825

[29] Azoulay E, Kouatchet A, Jaber S, et al. Noninvasive mechanical ventilation in patients having declined tracheal intubation. Intensive Care Med. 2013;39(2):292–301. 23184037 10.1007/s00134-012-2746-2

[30] Azoulay E, Resche-Rigon M, Megarbane B, et al. Association of COVID-19 acute respiratory distress syndrome with symptoms of posttraumatic stress disorder in family members after ICU discharge. JAMA. 2022;327(11):1042–50. 35179564 10.1001/jama.2022.2017 PMC8924722

[31] Bannon S, Lester EG, Gates MV, et al. Recovering together: building resiliency in dyads of stroke patients and their caregivers at risk for chronic emotional distress; a feasibility study. Pilot Feasibility Stud. 2020;6:75. 32509320 10.1186/s40814-020-00615-z PMC7249683

[32] Beesley SJ, Hopkins RO, Holt-Lunstad J, et al. Acute physiologic stress and subsequent anxiety among family members of ICU patients. Crit Care Med. 2018;46(2):229–35. 29112079 10.1097/CCM.0000000000002835 PMC5770106

[33] Beesley SJ, Hirshberg EL, Wilson EL, et al. Depression and change in caregiver burden among family members of intensive care unit survivors. Am J Crit Care. 2020;29(5):350–7. 32869070 10.4037/ajcc2020181

[34] Harris BR, Beesley SJ, Hopkins RO, et al. Heart rate variability and subsequent psychological distress among family members of intensive care unit patients. J Int Med Res. 2021;49(11):3000605211057829. 34846178 10.1177/03000605211057829 PMC8649465

[35] Bohart S, Egerod I, Bestle MH, Overgaard D, Christensen DF, Jensen JF. Recovery programme for ICU survivors has no effect on relatives’ quality of life: secondary analysis of the RAPIT-study. Intensive Crit Care Nurs. 2018;47:39–45. 29606480 10.1016/j.iccn.2018.03.002

[36] Carson SS, Cox CE, Wallenstein S, et al. Effect of palliative care-led meetings for families of patients with chronic critical illness: a randomized clinical trial. JAMA. 2016;316(1):51–62. 27380343 10.1001/jama.2016.8474 PMC5538801

[37] Cattelan J, Castellano S, Merdji H, et al. Psychological effects of remote-only communication among reference persons of ICU patients during COVID-19 pandemic. J Intensive Care. 2021;9(1):5. 33422153 10.1186/s40560-020-00520-w PMC7794617

[38] Cox CE, Hough CL, Carson SS, et al. Can coping-skills training help patients who have received intensive hospital care to cope with depression and anxiety? Washington (DC): Patient-Centered Outcomes Research Institute (PCORI); 2018. Available from: https://www.ncbi.nlm.nih.gov/books/NBK591018/37079709

[39] de Miranda S, Pochard F, Chaize M, et al. Postintensive care unit psychological burden in patients with chronic obstructive pulmonary disease and informal caregivers: a multicenter study. Crit Care Med. 2011;39(1):112–8. 21037472 10.1097/CCM.0b013e3181feb824

[40] de Ridr C, Zegers M, Jagernath D, Brunnekreef G, van den Boogaard M. Psychological symptoms in relatives of critically ill patients: a longitudinal cohort study. Crit Care Explor. 2021;3(7):e0470. 34235457 10.1097/CCE.0000000000000470 PMC8238357

[41] Dijkstra BM, Rood PJT, Teerenstra S, et al. Effect of a standardized family participation program in the ICU: a multicenter stepped-wedge cluster randomized controlled trial. Crit Care Med. 2024;52(3):420–31. 37934138 10.1097/CCM.0000000000006093 PMC10876177

[42] Fumis RRL, Ranzani OT, Martins PS, Schettino G. Emotional disorders in pairs of patients and their family members during and after ICU stay. PLoS ONE. 2015;10(1):e0115332. 25616059 10.1371/journal.pone.0115332 PMC4304779

[43] Fumis RRL, Ferraz AB, de Castro I, de Barros Oliveira HS, Moock M, Junior JMV. Mental health and quality of life outcomes in family members of patients with chronic critical illness admitted to the intensive care units of two Brazilian hospitals serving the extremes of the socioeconomic spectrum. PLoS ONE. 2019;14(9):e0221218. 31518359 10.1371/journal.pone.0221218 PMC6743763

[44] Garrouste-Orgeas M, Coquet I, Perier A, et al. Impact of an intensive care unit diary on psychological distress in patients and relatives*. Crit Care Med. 2012;40(7):2033–40. 22584757 10.1097/CCM.0b013e31824e1b43

[45] Garrouste-Orgeas M, Max A, Lerin T, et al. Impact of proactive nurse participation in ICU family conferences: a mixed-method study. Crit Care Med. 2016;44(6):1116–28. 26937860 10.1097/CCM.0000000000001632

[46] Garrouste-Orgeas M, Flahault C, Vinatier I, et al. Effect of an ICU diary on posttraumatic stress disorder symptoms among patients receiving mechanical ventilation: a randomized clinical trial. JAMA. 2019;322(3):229–39. 31310299 10.1001/jama.2019.9058 PMC6635906

[47] Gonzalez-Martin S, Becerro-de-Bengoa-Vallejo R, Rodriguez-Garcia M, et al. Influence on depression, anxiety, and satisfaction of the relatives’ visit to intensive care units prior to hospital admission for elective cardiac surgery: a randomized clinical trial. Int J Clin Pract. 2022;2022:1746782. 35685601 10.1155/2022/1746782 PMC9159139

[48] Greenleaf B, Foy A, Van Scoy L. Relationships between personality traits and perceived stress in surrogate decision-makers of intensive care unit patients. Am J Hosp Palliat Care. 2024;41(6):664–72. 37641412 10.1177/10499091231197662 PMC11032632

[49] Harlan EA, Miller J, Costa DK, et al. Emotional experiences and coping strategies of family members of critically ill patients. Chest. 2020;158(4):1464–72. 32454044 10.1016/j.chest.2020.05.535 PMC7545490

[50] Heesakkers H, van der Hoeven JG, Corsten S, et al. Mental health symptoms in family members of COVID-19 ICU survivors 3 and 12 months after ICU admission: a multicentre prospective cohort study. Intensive Care Med. 2022;48(3):322–31. 35103824 10.1007/s00134-021-06615-8 PMC8804080

[51] Henderson P, Quasim T, Asher A, et al. Post-intensive care syndrome following cardiothoracic critical care: feasibility of a complex intervention. J Rehabil Med. 2021;53(6):jrm00206. 33856038 10.2340/16501977-2825 PMC8814889

[52] Hickman RL, Pignatiello GA, Tahir S. Evaluation of the decisional fatigue scale among surrogate decision makers of the critically ill. West J Nurs Res. 2018;40(2):191–208. 28805132 10.1177/0193945917723828 PMC5750078

[53] Kentish-Barnes N, Azoulay E, Reignier J, et al. A randomised controlled trial of a nurse facilitator to promote communication for family members of critically ill patients. Intensive Care Med. 2024;50(5):712–24. 38573403 10.1007/s00134-024-07390-y

[54] Komachi MH, Kamibeppu K. Posttraumatic stress symptoms in families of cancer patients admitted to the intensive care unit: a longitudinal study. J Intensive Care. 2016;4:47. 27446590 10.1186/s40560-016-0162-3 PMC4955156

[55] Lester EG, Silverman IH, Gates MV, Lin A, Vranceanu A-M. Associations between gender, resiliency factors, and anxiety in Neuro-ICU caregivers: a prospective study. Int J Behav Med. 2020;27(6):677–86. 32488793 10.1007/s12529-020-09907-3

[56] Matt B, Schwarzkopf D, Reinhart K, Konig C, Hartog CS. Relatives’ perception of stressors and psychological outcomes—results from a survey study. J Crit Care. 2017;39:172–7. 28273613 10.1016/j.jcrc.2017.02.036

[57] McAdam JL, Fontaine DK, White DB, Dracup KA, Puntillo KA. Psychological symptoms of family members of high-risk intensive care unit patients. Am J Crit Care. 2012;21(6):386–94. 23117902 10.4037/ajcc2012582

[58] Meyers EE, Presciutti A, Shaffer KM, et al. The impact of resilience factors and anxiety during hospital admission on longitudinal anxiety among dyads of neurocritical care patients without major cognitive impairment and their family caregivers. Neurocrit Care. 2020;33(2):468–78. 31997141 10.1007/s12028-020-00913-7 PMC12054369

[59] Meyers EE, Shaffer KM, Gates M, Lin A, Rosand J, Vranceanu A-M. Baseline resilience and posttraumatic symptoms in dyads of neurocritical patients and their informal caregivers: a prospective dyadic analysis. Psychosomatics. 2020;61(2):135–44. 31928783 10.1016/j.psym.2019.11.007 PMC7035969

[60] Meyers E, Lin A, Lester E, Shaffer K, Rosand J, Vranceanu A-M. Baseline resilience and depression symptoms predict trajectory of depression in dyads of patients and their informal caregivers following discharge from the Neuro-ICU. Gen Hosp Psychiatry. 2020;62:87–92. 31887641 10.1016/j.genhosppsych.2019.12.003 PMC6948176

[61] Milton A, Schandl A, Larsson I-M, et al. Caregiver burden and emotional wellbeing in informal caregivers to ICU survivors-a prospective cohort study. Acta Anaesthesiol Scand. 2022;66(1):94–102. 34582048 10.1111/aas.13988

[62] de Oliveira HSB, Fumis RRL. Sex and spouse conditions influence symptoms of anxiety, depression, and posttraumatic stress disorder in both patients admitted to intensive care units and their spouses. Rev Bras Ter Intensiva. 2018;30(1):35–41. 29742213 10.5935/0103-507X.20180004 PMC5885229

[63] Petrinec AB, Mazanec PM, Burant CJ, Hoffer A, Daly BJ. Coping strategies and posttraumatic stress symptoms in post-ICU family decision makers. Crit Care Med. 2015;43(6):1205–12. 25785520 10.1097/CCM.0000000000000934 PMC4818005

[64] Petrinec A. Post-intensive care syndrome in family decision makers of long-term acute care hospital patients. Am J Crit Care. 2017;26(5):416–22. 28864439 10.4037/ajcc2017414

[65] Petrinec AB, Martin BR. Post-intensive care syndrome symptoms and health-related quality of life in family decision-makers of critically ill patients. Palliat Support Care. 2018;16(6):719–24. 29277171 10.1017/S1478951517001043

[66] Petrinec A, Wilk C, Hughes JW, Zullo MD, Chen Y-J, Palmieri PA. Delivering cognitive behavioral therapy for post-intensive care syndrome-family via a mobile health app. Am J Cri Care. 2021;30(6):451–8. 10.4037/ajcc2021962 34719716

[67] Petrinec AB, Wilk C, Hughes JW, Zullo MD, George RL. Self-care mental health app intervention for post-intensive care syndrome-family: a randomized pilot study. Am J Crit Care. 2023;32(6):440–8. 37907376 10.4037/ajcc2023800

[68] Torres J, Carvalho D, Molinos E, et al. The impact of the patient post-intensive care syndrome components upon caregiver burden. Med Intensiva. 2017;41(8):454–60. 28188064 10.1016/j.medin.2016.12.005

[69] van Veenendaal N, van der Meulen IC, Onrust M, Paans W, Dieperink W, van der Voort PHJ. Six-month outcomes in COVID-19 ICU patients and their family members: a prospective cohort study. Healthcare (Basel). 2021;9(7):865. 34356243 10.3390/healthcare9070865 PMC8305246

[70] Veislinger-Burelli G, Vincent A, Mallard J, et al. Impact of a visual support dedicated to prognosis on symptoms of stress of ICU family members: a before-and-after implementation study. Crit Care Explor. 2021;3(7):e0483. 34278313 10.1097/CCE.0000000000000483 PMC8280076

[71] Viana DDR, Santana LB, Azzolin KO, et al. Quality of life and satisfaction of relatives of patients admitted to intensive care units. Cogitare Enferm. 2023;28:e93169.

[72] Vranceanu A-M, Bannon S, Mace R, et al. Feasibility and efficacy of a resiliency intervention for the prevention of chronic emotional distress among survivor-caregiver dyads admitted to the Neuroscience Intensive Care Unit: a randomized clinical trial. JAMA Netw Open. 2020;3(10):e2020807. 33052404 10.1001/jamanetworkopen.2020.20807 PMC7557506

[73] Wiertz CMH, Hemmen B, Sep SJS, Verbunt JA. Caregiver burden and impact on COVID-19 patient participation and quality of life one year after ICU discharge—a prospective cohort study. Patient Educ Couns. 2024;123:108221. 38460347 10.1016/j.pec.2024.108221

[74] Zante B, Erne K, Grossenbacher J, Camenisch SA, Schefold JC, Jeitziner M-M. Symptoms of post-traumatic stress disorder (PTSD) in next of kin during suspension of ICU visits during the COVID-19 pandemic: a prospective observational study. BMC Psychiatry. 2021;21(1):477. 34587929 10.1186/s12888-021-03468-9 PMC8480126

[75] Lautrette A, Darmon M, Megarbane B, et al. A communication strategy and brochure for relatives of patients dying in the ICU. N Engl J Med. 2007;356(5):469–78. 17267907 10.1056/NEJMoa063446

[76] Academia EC, Gabriel CJ, Mueller A, et al. Opioid prescribing after discharge in a previously mechanically ventilated, opioid-naïve cohort. Ann Pharmacother. 2020;54(11):1065–72. 32349532 10.1177/1060028020919122

[77] Higgins JP, Thompson SG, Spiegelhalter DJ. A re-evaluation of random-effects meta-analysis. J R Stat Soc Ser A Stat Soc. 2009;172(1):137–59. 19381330 10.1111/j.1467-985X.2008.00552.x PMC2667312

[78] Lobato CT, Camoes J, Carvalho D, et al. Risk factors associated with post-intensive care syndrome in family members (PICS-F): a prospective observational study. J Intensive Care Soc. 2023;24(3):247–57. 37744068 10.1177/17511437221108904 PMC10515326

