# Supplementary material for: Anxiety, depression and distress in family members of people who have experienced a critical care admission: a systematic review and Bayesian meta-analysis
Source: J Intensive Care. 2025 Dec 15;14:3. doi: 10.1186/s40560-025-00839-2 (PMC12771835; doi:10.1186/s40560-025-00839-2)
Supplement: Supplementary file 1 — Additional file 1. [file 40560_2025_839_MOESM1_ESM.docx]

**Supplementary Materials**

**e-Table 1. Summary of search results**

| Medline via Ovid | 5192 |
| --- | --- |
| PsycINFO via EbscoHost | 1151 |
| CINAHL via EbscoHost | 4866 |
| Web of Science Core Collection | 3936 |
| Scopus | 4813 |
| Total | 19958 |
| No. duplicates removed | 10021 |
| Total unique hits | 9937 |

**e-Table 2. Medline search strategy**

Ovid MEDLINE(R) and Epub Ahead of Print, In-Process, In-Data-Review & Other Non-Indexed Citations, Daily and Versions <1946 to May 03, 2024>

| **#** | **Query** | **Results** |
| --- | --- | --- |
| 1 | ("mental health" or "psychological symptom*" or "psychological stress" or "mental stress*" or "depression" or anxiety or PTSD or "post traumatic stress" or "posttraumatic stress" or "traumatic stress" or employ* or unemploy* or "social work" or welfare or strain or burden* or burnout or "burn* out").ti,ab,kw,kf. | 2498980 |
| 2 | exp Mental Health/ | 67133 |
| 3 | exp Stress, Psychological/ | 156348 |
| 4 | exp Depression/ | 157422 |
| 5 | exp Anxiety Disorders/ or exp Anxiety/ | 195739 |
| 6 | exp Stress Disorders, Post-Traumatic/ | 42985 |
| 7 | exp Employment/ | 102276 |
| 8 | exp Unemployment/ | 7902 |
| 9 | exp Psychological Distress/ | 7376 |
| 10 | exp Social Work/ | 18880 |
| 11 | exp Social Welfare/ | 61609 |
| 12 | exp Symptom Burden/ | 55 |
| 13 | exp Caregiver Burden/ | 736 |
| 14 | or/1-13 | 2790540 |
| 15 | ("critical* ill*" or "intensive care" or "critical care" or ICU or CCU or "high dependency").ti,ab,kw,kf. | 302773 |
| 16 | exp Critical Care/ | 68150 |
| 17 | exp Critical Illness/ | 40491 |
| 18 | exp Intensive Care Units/ | 109423 |
| 19 | (sepsis or septic or septic?em* or "acute respiratory failure" or "acute respiratory insufficient*" or "respiratory distress" or "acute respiratory distress" or "pulmonary distress" or "lung distress").ti,ab,kw,kf. | 246882 |
| 20 | exp Sepsis/ | 145587 |
| 21 | exp Shock, Septic/ | 25496 |
| 22 | exp Respiratory Distress Syndrome/ | 41394 |
| 23 | or/15-22 | 630968 |
| 24 | (family or families or relatives or caregiver* or care giver* or carer* or spouse* or proxy or proxies or "patient agent*").ti,ab,kw,kf. | 1366961 |
| 25 | exp Family/ | 377525 |
| 26 | exp Caregivers/ | 52912 |
| 27 | exp Spouses/ | 11803 |
| 28 | exp Proxy/ | 1826 |
| 29 | or/24-28 | 1600129 |
| 30 | 14 and 23 and 29 | 5982 |
| 31 | limit 30 to yr="2000 -Current" | 5192 |

**e-Table 3. PsycINFO**

| **#** | **Query** | **Results** |
| --- | --- | --- |
| S1 | TI("mental health" or "psychological symptom*" or “psychological stress” or “mental stress*” or "depression" or anxiety or PTSD or "post traumatic stress" or "posttraumatic stress" or “traumatic stress” or employ* or unemploy* or "social work" or welfare or strain or burden* or burnout or “burn* out”) or AB("mental health" or "psychological symptom*" or “psychological stress” or “mental stress*” or "depression" or anxiety or PTSD or "post traumatic stress" or "posttraumatic stress" or “traumatic stress” or employ* or unemploy* or "social work" or welfare or strain or burden* or burnout or “burn* out”) | 1,020,653 |
| S2 | DE "Mental Health" OR DE "Athlete Mental Health" OR DE "Mental Health Parity" OR DE "Military Mental Health" OR DE "Youth Mental Health" | 103,887 |
| S3 | DE "Major Depression" OR DE "Anaclitic Depression" OR DE "Dysthymic Disorder" OR DE "Endogenous Depression" OR DE "Late Life Depression" OR DE "Postpartum Depression" OR DE "Reactive Depression" OR DE "Recurrent Depression" OR DE "Seasonal Affective Disorder" OR DE "Treatment Resistant Depression" | 169,267 |
| S4 | DE "Anxiety" OR DE "Anxiety Sensitivity" OR DE "Climate Anxiety" OR DE "Computer Anxiety" OR DE "Death Anxiety" OR DE "Health Anxiety" OR DE "Mathematics Anxiety" OR DE "Performance Anxiety" OR DE "Social Anxiety" OR DE "Speech Anxiety" OR DE "Test Anxiety" OR DE "Travel Anxiety" OR DE "Anxiety Disorders" OR DE "Castration Anxiety" OR DE "Generalized Anxiety Disorder" OR DE "Panic Attack" OR DE "Panic Disorder" OR DE "Phobias" OR DE "Selective Mutism" OR DE "Separation Anxiety Disorder" | 143,740 |
| S5 | DE "Anxiety" OR DE "Anxiety Sensitivity" OR DE "Climate Anxiety" OR DE "Computer Anxiety" OR DE "Death Anxiety" OR DE "Health Anxiety" OR DE "Mathematics Anxiety" OR DE "Performance Anxiety" OR DE "Social Anxiety" OR DE "Speech Anxiety" OR DE "Test Anxiety" OR DE "Travel Anxiety" OR DE "Anxiety Disorders" OR DE "Castration Anxiety" OR DE "Generalized Anxiety Disorder" OR DE "Panic Attack" OR DE "Panic Disorder" OR DE "Phobias" OR DE "Selective Mutism" OR DE "Separation Anxiety Disorder" | 143,740 |
| S6 | DE "Posttraumatic Stress Disorder" OR DE "Complex PTSD" | 42,322 |
| S7 | DE "Psychological Stress" | 9,115 |
| S8 | DE "Employment Status" OR DE "Employability" OR DE "Employment History" OR DE "Job Loss" OR DE "Reemployment" OR DE "Retirement" OR DE "Self-Employment" OR DE "Unemployment" | 33,817 |
| S9 | (DE "Social Workers" OR DE "Psychiatric Social Workers") OR (DE "Welfare Services (Government)") | 18,793 |
| S10 | DE "Caregiver Burden" or DE "Burnout" OR DE "Academic Stress" OR DE "Athletic Burnout" OR DE "Occupational Stress" | 35,959 |
| S11 | S1 OR S2 OR S3 OR S4 OR S5 OR S6 OR S7 OR S8 OR S9 OR S10 | 1,099,592 |
| S12 | TI("critical* ill*" or "intensive care" or "critical care" or ICU or CCU or "high dependency") or AB("critical* ill*" or "intensive care" or "critical care" or ICU or CCU or "high dependency") | 13,724 |
| S13 | (DE "Critical Illness") OR (DE "Intensive Care" OR DE "Neonatal Intensive Care") | 8,112 |
| S14 | TI(sepsis or septic or septic?em* or "acute respiratory failure" or “acute respiratory insufficient*” or “respiratory distress” or “acute respiratory distress” or “pulmonary distress” or “lung distress”) or AB(sepsis or septic or septic?em* or "acute respiratory failure" or “acute respiratory insufficient*” or “respiratory distress” or “acute respiratory distress” or “pulmonary distress” or “lung distress”) | 2,065 |
| S15 | (DE "Sepsis") OR (DE "Respiratory Distress" OR DE "Apnea" OR DE "Dyspnea" OR DE "Hyperventilation") | 3,020 |
| S16 | S12 OR S13 OR S14 OR S15 | 19,232 |
| S17 | TI(family or families or relatives or caregiver* or "care giver*" or carer* or spouse* or proxy or proxies or “patient agent*” ) or AB(family or families or relatives or caregiver* or "care giver*" or carer* or spouse* or proxy or proxies or “patient agent*” ) | 650,329 |
| S18 | DE "Family" OR DE "Biological Family" OR DE "Dual Careers" OR DE "Dysfunctional Family" OR DE "Extended Family" OR DE "Family Background" OR DE "Family History" OR DE "Family Members" OR DE "Family of Origin" OR DE "Family Relations" OR DE "Family Resemblance" OR DE "Family Structure" OR DE "Family Work Relationship" OR DE "Interethnic Family" OR DE "Interracial Family" OR DE "Marriage" OR DE "Military Families" OR DE "Nepotism" OR DE "Nuclear Family" OR DE "Offspring" OR DE "Stepfamily" | 165,815 |
| S19 | MM "Caregivers" | 29,900 |
| S20 | DE "Spouses" OR DE "Husbands" OR DE "Wives" | 17,845 |
| S21 | S17 OR S18 OR S19 OR S20 | 683,266 |
| S22 | S11 AND S16 AND S21 | 1,262 |
| S23 | S11 AND S16 AND S21 | 1,150 |

**e-Table 4. CINAHL**

| **#** | **Query** | **Results** |
| --- | --- | --- |
| S1 | TI("mental health" or "psychological symptom*" or “psychological stress” or “mental stress*” or "depression" or anxiety or PTSD or "post traumatic stress" or "posttraumatic stress" or “traumatic stress” or employ* or unemploy* or "social work" or welfare or strain or burden* or burnout or “burn* out”) or AB("mental health" or "psychological symptom*" or “psychological stress” or “mental stress*” or "depression" or anxiety or PTSD or "post traumatic stress" or "posttraumatic stress" or “traumatic stress” or employ* or unemploy* or "social work" or welfare or strain or burden* or burnout or “burn* out”) | 657,733 |
| S2 | (MH "Mental Health") OR (MH "Stress, Psychological+") OR (MH "Stress+") OR (MH "Depression+") OR (MH "Anxiety+") OR (MH "Anxiety Disorders+") OR (MH "Stress Disorders, Post-Traumatic+") | 352,953 |
| S3 | (MH "Employment+") OR (MH "Employment Status") OR (MH "Unemployment") OR (MH "Social Welfare+") OR (MH "Social Workers") OR (MH "Social Work+") OR (MH "Caregiver Burden") OR (MH "Symptom Burden") OR (MH "Caregiver Support") | 149,527 |
| S4 | S1 OR S2 OR S3 | 884,927 |
| S5 | TI("critical* ill*" or "intensive care" or "critical care" or ICU or CCU or "high dependency") or AB("critical* ill*" or "intensive care" or "critical care" or ICU or CCU or "high dependency") | 130,513 |
| S6 | (MH "Critical Care+") OR (MH "Intensive Care Units+") OR (MH "Critically Ill Patients") OR (MH "Critical Illness") | 112,230 |
| S7 | TI(sepsis or septic or septic?em* or "acute respiratory failure" or “acute respiratory insufficient*” or “respiratory distress” or “acute respiratory distress” or “pulmonary distress” or “lung distress”) or AB(sepsis or septic or septic?em* or "acute respiratory failure" or “acute respiratory insufficient*” or “respiratory distress” or “acute respiratory distress” or “pulmonary distress” or “lung distress”) | 52,381 |
| S8 | (MH "Sepsis+") OR (MH "Shock, Septic+") OR (MH "Respiratory Distress Syndrome, Acute") | 40,427 |
| S9 | S5 OR S6 OR S7 OR S8 | 220,759 |
| S10 | TI(family or families or relatives or caregiver* or "care giver*" or carer* or spouse* or proxy or proxies or “patient agent*” ) or AB(family or families or relatives or caregiver* or "care giver*" or carer* or spouse* or proxy or proxies or “patient agent*” ) | 508,732 |
| S11 | (MH "Family+") OR (MH "Extended Family+") OR (MH "Spouses") OR (MH "Caregivers") OR (MH "Proxy") | 306,558 |
| S12 | S10 OR S11 | 682,970 |
| S13 | S4 AND S9 AND S12 | 5,236 |
| S14 | S4 AND S9 AND S12 | 4,866 |

**e-Table 5. Scopus**

| **#** | **Query** | **Results** |
| --- | --- | --- |
|  | TITLE-ABS ( ( "mental health" OR "psychological symptom*" OR "psychological stress" OR "mental stress*" OR "depression" OR anxiety OR ptsd OR "post traumatic stress" OR "posttraumatic stress" OR "traumatic stress" OR employ* OR unemploy* OR "social work" OR welfare OR strain OR burden* OR burnout OR "burn* out" ) ) AND TITLE-ABS ( ( "critical* ill*" OR "intensive care" OR "critical care" OR icu OR ccu OR "high dependency" ) OR ( sepsis OR septic OR septic?em* OR "acute respiratory failure" OR "acute respiratory insufficient*" OR "respiratory distress" OR "acute respiratory distress" OR "pulmonary distress" OR "lung distress" ) ) AND TITLE-ABS ( family OR families OR relatives OR caregiver* OR "care giver*" OR carer* OR spouse* OR proxy OR proxies OR "patient agent*" ) AND PUBYEAR > 2000 AND PUBYEAR < 2025 | 4813 |

**e-Table 6. Web of Science Core Collection**

# Entitlements:

- WOS.IC: 1993 to 2024

- WOS.CCR: 1985 to 2024

- WOS.SCI: 1900 to 2024

- WOS.AHCI: 1975 to 2024

- WOS.BHCI: 2008 to 2024

- WOS.BSCI: 2008 to 2024

- WOS.ESCI: 2019 to 2024

- WOS.ISTP: 1990 to 2024

- WOS.SSCI: 1956 to 2024

- WOS.ISSHP: 1990 to 2024

| **#** | **Query** | **Results** |
| --- | --- | --- |
| **1** | TI=("mental health" or "psychological symptom*" or “psychological stress” or “mental stress*” or "depression" or anxiety or PTSD or "post traumatic stress" or "posttraumatic stress" or “traumatic stress” or employ* or unemploy* or "social work" or welfare or strain or burden* or burnout or “burn* out”) or AB=("mental health" or "psychological symptom*" or “psychological stress” or “mental stress*” or "depression" or anxiety or PTSD or "post traumatic stress" or "posttraumatic stress" or “traumatic stress” or employ* or unemploy* or "social work" or welfare or strain or burden* or burnout or “burn* out”) | 5051185 |
| **2** | TI=("critical* ill*" or "intensive care" or "critical care" or ICU or CCU or "high dependency") or AB=("critical* ill*" or "intensive care" or "critical care" or ICU or CCU or "high dependency") | 285580 |
| **3** | TI=(sepsis or septic or septic?em* or "acute respiratory failure" or “acute respiratory insufficient*” or “respiratory distress” or “acute respiratory distress” or “pulmonary distress” or “lung distress”) or AB=(sepsis or septic or septic?em* or "acute respiratory failure" or “acute respiratory insufficient*” or “respiratory distress” or “acute respiratory distress” or “pulmonary distress” or “lung distress”) | 210712 |
| **4** | #2 OR #3 | 459412 |
| **5** | TI=(family or families or relatives or caregiver* or care giver* or carer* or spouse* or proxy or proxies or “patient agent*” ) or AB=(family or families or relatives or caregiver* or care giver* or carer* or spouse* or proxy or proxies or “patient agent*” ) | 3780743 |
| **6** | #1 AND #4 AND #5 | 4141 |
| **7** | #1 AND #4 AND #5 Timespan: 2000-01-01 to 2024-12-31 | 3936 |

**e-Table 7. Model Specifications**

|  | **Likelihood** | **Priors** | | | |
| --- | --- | --- | --- | --- | --- |
|  |  | **Pooled average** | **Between-study variability (τ)** | **Covariate** | **Sensitivity – pooled average** |
| Primary models (weighted average) | Gaussian (log link) | Normal(mean = log(midpoint*), SD spans full scale) | Half-Cauchy(location = 0, scale ≈ ½ scale range) | Not applicable | Normal(mean = log(1), SD spans full scale) |
| Meta-regression models (bereavement impact) | Gaussian (log link) | Normal(mean = log(midpoint*), SD spans full scale) | Half-Cauchy(location = 0, scale ≈ ½ scale range) | Normal(mean = 0, SD = 1) | n/a |
| HADS threshold models | Gaussian (log-odds) | Normal(mean = 0 [i.e. proportion of 50%], SD = 4) | Half-Cauchy(location = 0, scale = 5) | Not applicable | Normal(mean = –9 [i.e. proportion ~0%], SD = 4) |

*Midpoints for IES-R = 44, HADS-A and HADS-D = 10

**e-Table 8: IES-R Risk of Bias Assessment Summaries**

| **Study ID(s)** | **4.1. Is the sampling strategy relevant to address the research question?** | **4.2. Is the sample representative of the target population?** | **4.3. Are the measurements appropriate?** | **4.4. Is the risk of nonresponse bias low?** | **4.5. Is the statistical analysis appropriate to answer the research question?** |
| --- | --- | --- | --- | --- | --- |
| Azoulay, et al. ^1^ | Yes | Yes | Yes | Yes | Yes |
| Carson, et al. ^2^ | Yes | Yes | Yes | Yes | Yes |
| Cattelan, et al. ^3^ | Yes | Yes | Yes | No | Yes |
| Cox, et al. ^4^,  Cox, et al. ^5^ | Yes | Yes | Yes | Yes | Yes |
| de Miranda, et al. ^6^ | Yes | Yes | Yes | No | Yes |
| de Ridder, et al. ^7^ | Yes | Yes | Yes | Yes | Yes |
| Dijkstra, et al. ^8^ | Yes | Yes | Yes | Yes | Yes |
| Garrouste-Orgeas, et al. ^9^ | Yes | Yes | Yes | Yes | Yes |
| Garrouste-Orgeas, et al. ^10^ | Yes | Yes | Yes | Yes | Yes |
| Gonzalez-Martin, et al. ^11^ | Yes | Yes | Yes | Yes | Yes |
| Greenleaf, et al. ^12^ | Yes | Yes | Yes | No | Yes |
| Komachi, Kamibeppu ^13^ | Yes | Yes | Yes | No | Yes |
| McAdam, et al. ^14^ | Yes | Yes | Yes | Yes | Yes |
| Petrinec, et al. ^15^ | Yes | Yes | Yes | No | Yes |
| Zante, et al. ^16^ | Yes | Yes | Yes | Yes | Yes |

**e-Table 9: HADS-A Risk of Bias Assessment Summaries**

| **Study ID(s)** | 4.1. Is the sampling strategy relevant to address the research question? | 4.2. Is the sample representative of the target population? | 4.3. Are the measurements appropriate? | 4.4. Is the risk of nonresponse bias low? | 4.5. Is the statistical analysis appropriate to answer the research question? |
| --- | --- | --- | --- | --- | --- |
| Amass, et al. ^17^,  Amass, et al. ^18^ | Yes | Yes | Yes | No | Yes |
| Amass, et al. ^19^ | Yes | Yes | Yes | Yes | Yes |
| Azoulay, et al. ^20^ | Yes | Yes | Yes | No | Yes |
| Azoulay, et al. ^1^ | Yes | Yes | Yes | Yes | Yes |
| Bannon, et al. ^21^ | Yes | No | Yes | No | Yes |
| Beesley, et al. ^22^,  Beesley, et al. ^23^  Harris, et al. ^24^ | Yes | Yes | Yes | Yes | Yes |
| Bohart, et al. ^25^ | Yes | Yes | Yes | Yes | No |
| Carson, et al. ^2^ | Yes | Yes | Yes | Yes | Yes |
| Cox 2018 | Yes | Yes | Yes | Yes | Yes |
| de Miranda, et al. ^6^ | Yes | Yes | Yes | No | Yes |
| de Ridder, et al. ^7^ | Yes | Yes | Yes | Yes | Yes |
| Dijkstra, et al. ^8^ | Yes | Yes | Yes | Yes | Yes |
| Fumis, et al. ^26^ | Yes | Yes | Yes | No | Yes |
| Fumis, et al. ^27^ | Yes | Yes | Yes | No | Yes |
| Garrouste-Orgeas, et al. ^28^ | Yes | Yes | Yes | Yes | Yes |
| Garrouste-Orgeas, et al. ^9^ | Yes | Yes | Yes | Yes | Yes |
| Garrouste-Orgeas, et al. ^10^ | Yes | Yes | Yes | Yes | Yes |
| Gonzalez-Martin, et al. ^11^ | Yes | Yes | Yes | Yes | Yes |
| Harlan, et al. ^29^ | Yes | Yes | Yes | No | Yes |
| Heesakkers, et al. ^30^ | Yes | Yes | Yes | No | Yes |
| Henderson, et al. ^31^ | Yes | Yes | Yes | Yes | Yes |
| Hickman, et al. ^32^ | Yes | Yes | Yes | Yes | Yes |
| Kentish-Barnes, et al. ^33^ | Yes | Yes | Yes | No | Yes |
| Lester, et al. ^34^ | Yes | Yes | Yes | Yes | Yes |
| Lobato, et al. ^35^ | Yes | Yes | Yes | No | Yes |
| Matt, et al. ^36^ | Yes | Yes | Yes | No | Yes |
| McAdam, et al. ^14^ | Yes | Yes | Yes | Yes | Yes |
| Meyers, et al. ^37^,  Meyers, et al. ^38^,  Meyers, et al. ^39^ | Yes | Yes | Yes | No | Yes |
| Milton, et al. ^40^ | Yes | Yes | Yes | Yes | Yes |
| Oliveira, Fumis ^41^ | Yes | Yes | Yes | No | Yes |
| Petrinec ^42^ | Yes | Yes | Yes | Yes | Yes |
| Petrinec, Martin ^43^ | Yes | Yes | Yes | No | Yes |
| Petrinec, et al. ^44^ | Yes | Yes | Yes | Yes | Yes |
| Petrinec, et al. ^45^ | Yes | Yes | Yes | Yes | Yes |
| Torres, et al. ^46^ | Yes | Yes | Yes | Yes | No |
| van Veenendaal, et al. ^47^ | Yes | Yes | Yes | No | Yes |
| Veislinger-Burelli, et al. ^48^ | Yes | Yes | Yes | Yes | Yes |
| Viana, et al. ^49^ | Yes | Yes | Yes | No | Yes |
| Vranceanu, et al. ^50^ | Yes | Yes | Yes | Yes | Yes |
| Wiertz, et al. ^51^ | Yes | Yes | Yes | Yes | Yes |

**e-Table 10: HADS-D Risk of Bias Assessment Summaries**

| **Study ID(s)** | **4.1. Is the sampling strategy relevant to address the research question?** | **4.2. Is the sample representative of the target population?** | **4.3. Are the measurements appropriate?** | **4.4. Is the risk of nonresponse bias low?** | **4.5. Is the statistical analysis appropriate to answer the research question?** |
| --- | --- | --- | --- | --- | --- |
| Amass, et al. ^17^,  Amass, et al. ^18^ | Yes | Yes | Yes | No | Yes |
| Amass, et al. ^19^ | Yes | Yes | Yes | Yes | Yes |
| Azoulay, et al. ^20^ | Yes | Yes | Yes | No | Yes |
| Azoulay, et al. ^1^ | Yes | Yes | Yes | Yes | Yes |
| Bannon, et al. ^21^ | Yes | No | Yes | No | Yes |
| Beesley, et al. ^22^,  Beesley, et al. ^23^  Harris, et al. ^24^ | Yes | Yes | Yes | Yes | Yes |
| Bohart, et al. ^25^ | Yes | Yes | Yes | Yes | No |
| Carson, et al. ^2^ | Yes | Yes | Yes | Yes | Yes |
| Cinotti, et al. ^52^ | Yes | Yes | Yes | Yes | Yes |
| Cox, et al. ^4^,  Cox, et al. ^5^ | Yes | Yes | Yes | Yes | Yes |
| de Miranda, et al. ^6^ | Yes | Yes | Yes | No | No |
| de Ridder, et al. ^7^ | Yes | Yes | Yes | Yes | Yes |
| Dijkstra, et al. ^8^ | Yes | Yes | Yes | Yes | Yes |
| Fumis, et al. ^26^ | Yes | Yes | Yes | No | Yes |
| Fumis, et al. ^27^ | Yes | Yes | Yes | No | Yes |
| Garrouste-Orgeas, et al. ^28^ | Yes | Yes | Yes | Yes | Yes |
| Garrouste-Orgeas, et al. ^9^ | Yes | Yes | Yes | Yes | Yes |
| Garrouste-Orgeas, et al. ^10^ | Yes | Yes | Yes | Yes | Yes |
| Gonzalez-Martin, et al. ^11^ | Yes | Yes | Yes | Yes | Yes |
| Harlan, et al. ^29^ | Yes | Yes | Yes | No | No |
| Heesakkers, et al. ^30^ | Yes | Yes | Yes | No | Yes |
| Henderson, et al. ^31^ | Yes | Yes | Yes | Yes | Yes |
| Hickman, et al. ^32^ | Yes | Yes | Yes | Yes | Yes |
| Kentish-Barnes, et al. ^33^ | Yes | Yes | Yes | No | Yes |
| Lobato, et al. ^35^ | Yes | Yes | Yes | No | Yes |
| Matt, et al. ^36^ | Yes | Yes | Yes | No | Yes |
| McAdam, et al. ^14^ | Yes | Yes | Yes | Yes | Yes |
| Meyers, et al. ^37^,  Meyers, et al. ^38^,  Meyers, et al. ^39^ | Yes | Yes | Yes | No | Yes |
| Milton, et al. ^40^ | Yes | Yes | Yes | Yes | Yes |
| Oliveira, Fumis ^41^ | Yes | Yes | Yes | No | Yes |
| Petrinec ^42^ | Yes | Yes | Yes | Yes | Yes |
| Petrinec, Martin ^43^ | Yes | Yes | Yes | No | Yes |
| Petrinec, et al. ^44^ | Yes | Yes | Yes | Yes | Yes |
| Petrinec, et al. ^45^ | Yes | Yes | Yes | Yes | Yes |
| Torres, et al. ^46^ | Yes | Yes | Yes | Yes | No |
| Veislinger-Burelli, et al. ^48^ | Yes | Yes | Yes | Yes | Yes |
| Viana, et al. ^49^ | Yes | Yes | Yes | No | Yes |
| Vranceanu, et al. ^50^ | Yes | Yes | Yes | Yes | Yes |
| White, et al. ^53^ | Yes | Yes | Yes | No | Yes |
| Wiertz, et al. ^51^ | Yes | Yes | Yes | Yes | Yes |

**e-Table 11. MCMC Chain Convergence and Resolution**

| **Model** | **Variable** | **R̂** | **ESS Bulk** | **ESS Tail** |
| --- | --- | --- | --- | --- |
| IES-R primary model (weighted average) | Pooled Estimate | 1.00 | 11330.71 | 15757.64 |
| IES-R primary model (weighted average) | Between-study variance (τ) | 1.00 | 13365.70 | 21398.69 |
| IES-R conservative priors sensitivity analysis of primary model | Pooled Estimate | 1.00 | 10815.95 | 15766.04 |
| IES-R conservative priors sensitivity analysis of primary model | Between-study variance (τ) | 1.00 | 13000.63 | 20557.24 |
| IES-R no RCTs sensitivity analysis of primary model | Pooled Estimate | 1.00 | 13806.64 | 18649.26 |
| IES-R no RCTs sensitivity analysis of primary model | Between-study variance (τ) | 1.00 | 13325.87 | 18702.04 |
| IES-R low non-response sensitivity analysis of primary model | Pooled Estimate | 1.00 | 13454.77 | 17228.06 |
| IES-R low non-response sensitivity analysis of primary model | Between-study variance (τ) | 1.00 | 12936.54 | 18821.21 |
| HADS-A primary model (weighted average) | Pooled Estimate | 1.00 | 7717.9 | 15075.22 |
| HADS-A primary model (weighted average) | Between-study variance (τ) | 1.00 | 15084.88 | 24860.42 |
| HADS-A meta-regression model (bereavement impact) | Pooled Estimate | 1.00 | 10028.45 | 18223.99 |
| HADS-A meta-regression model (bereavement impact) | Moderator effect | 1.00 | 11557.16 | 19706.85 |
| HADS-A meta-regression model (bereavement impact) | Between-study variance (τ) | 1.00 | 15214.9 | 26830.21 |
| HADS-A conservative priors sensitivity analysis of primary model | Pooled Estimate | 1.00 | 5477.67 | 11733.13 |
| HADS-A conservative priors sensitivity analysis of primary model | Between-study variance (τ) | 1.00 | 14089.58 | 26114.43 |
| HADS-A no RCTs sensitivity analysis of primary model | Pooled Estimate | 1.00 | 8805.68 | 14621.75 |
| HADS-A no RCTs sensitivity analysis of primary model | Between-study variance (τ) | 1.00 | 13216.04 | 20796.45 |
| HADS-A low non-response sensitivity analysis of primary model | Pooled Estimate | 1.00 | 10487.54 | 17691.22 |
| HADS-A low non-response sensitivity analysis of primary model | Between-study variance (τ) | 1.00 | 16218.36 | 23959.92 |
| HADS-A threshold model | Pooled Estimate | 1.00 | 19840.19 | 29221.07 |
| HADS-A threshold model | Between-study variance (τ) | 1.00 | 20024.57 | 27981.81 |
| HADS-A conservative priors sensitivity analysis of threshold model | Pooled Estimate | 1.00 | 19343.67 | 26561.20 |
| HADS-A conservative priors sensitivity analysis of threshold model | Between-study variance (τ) | 1.00 | 18829.49 | 25764.52 |
| HADS-A no RCTs sensitivity analysis of threshold model | Pooled Estimate | 1.00 | 18001.24 | 24237.31 |
| HADS-A no RCTs sensitivity analysis of threshold model | Between-study variance (τ) | 1.00 | 17538 | 25650.2 |
| HADS-A low non-response sensitivity analysis of threshold model | Pooled Estimate | 1.00 | 16843.44 | 20865.51 |
| HADS-A low non-response sensitivity analysis of threshold model | Between-study variance (τ) | 1.00 | 14897.84 | 20439.24 |
| HADS-D primary model (weighted average) | Pooled Estimate | 1.00 | 11802.07 | 21356.31 |
| HADS-D primary model (weighted average) | Between-study variance (τ) | 1.00 | 16824.39 | 28672.73 |
| HADS-D meta-regression model (bereavement impact) | Pooled Estimate | 1.00 | 12658.3 | 22778.71 |
| HADS-D meta-regression model (bereavement impact) | Moderator effect | 1.00 | 16866.42 | 28473.28 |
| HADS-D meta-regression model (bereavement impact) | Between-study variance (τ) | 1.00 | 17099.24 | 27583.09 |
| HADS-D conservative priors sensitivity analysis of primary model | Pooled Estimate | 1.00 | 11500.07 | 21354.73 |
| HADS-D conservative priors sensitivity analysis of primary model | Between-study variance (τ) | 1.00 | 15812.64 | 29239.79 |
| HADS-D no RCTs sensitivity analysis of primary model | Pooled Estimate | 1.00 | 13382.25 | 23183.42 |
| HADS-D no RCTs sensitivity analysis of primary model | Between-study variance (τ) | 1.00 | 17201.43 | 26135.2 |
| HADS-D low non-response sensitivity analysis of primary model | Pooled Estimate | 1.00 | 12145.69 | 20976.2 |
| HADS-D low non-response sensitivity analysis of primary model | Between-study variance (τ) | 1.00 | 15511.89 | 23838.09 |
| HADS-D threshold model | Pooled Estimate | 1.00 | 28888.84 | 37444.56 |
| HADS-D threshold model | Between-study variance (τ) | 1.00 | 25713.24 | 34398.30 |
| HADS-D conservative priors sensitivity analysis of threshold model | Pooled Estimate | 1.00 | 28713.70 | 34106.16 |
| HADS-D conservative priors sensitivity analysis of threshold model | Between-study variance (τ) | 1.00 | 23998.42 | 36113.25 |
| HADS-D no RCTs sensitivity analysis of threshold model | Pooled Estimate |  | 27215.27 | 32658.95 |
| HADS-D no RCTs sensitivity analysis of threshold model | Between-study variance (τ) |  | 22916.8 | 30306.32 |
| HADS-D low non-response sensitivity analysis of threshold model | Pooled Estimate |  | 23015.23 | 25127.62 |
| HADS-D low non-response sensitivity analysis of threshold model | Between-study variance (τ) |  | 20347.04 | 21070.62 |

R̂ = potential scale reduction statistic, ESS = effective sample size

**e-Figure A. Posterior predictive checks for Bayesian models**


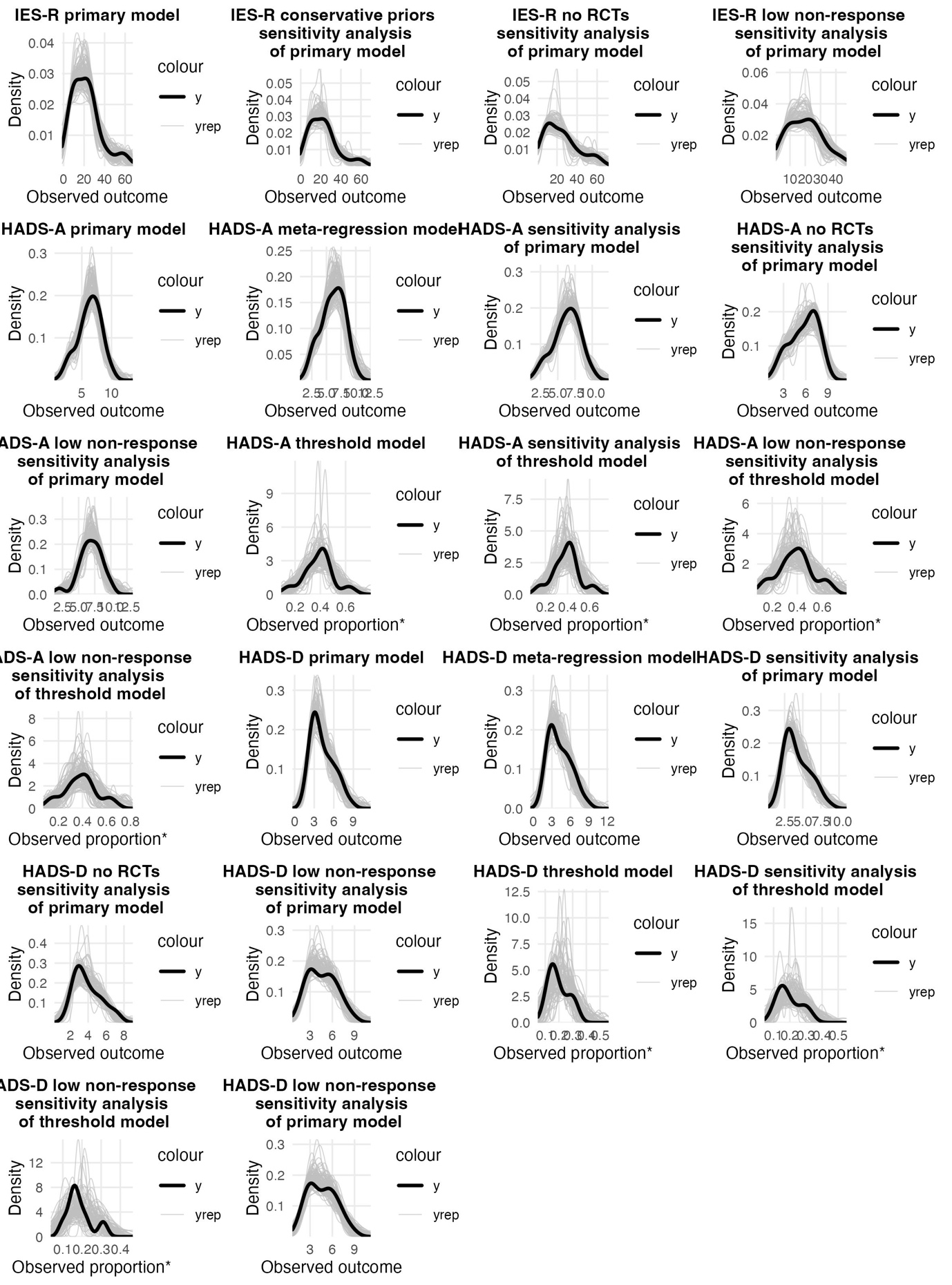


**References**

1. Azoulay E, Resche-Rigon M, Megarbane B, et al. Association of COVID-19 Acute Respiratory Distress Syndrome With Symptoms of Posttraumatic Stress Disorder in Family Members After ICU Discharge. *JAMA.* 2022;327(11):1042-1050.

2. Carson SS, Cox CE, Wallenstein S, et al. Effect of Palliative Care-Led Meetings for Families of Patients With Chronic Critical Illness: A Randomized Clinical Trial. *JAMA.* 2016;316(1):51-62.

3. Cattelan J, Castellano S, Merdji H, et al. Psychological effects of remote-only communication among reference persons of ICU patients during COVID-19 pandemic. *Journal of intensive care.* 2021;9(1):5.

4. Cox CE, Hough CL, Carson SS, et al. Effects of a Telephone- and Web-based Coping Skills Training Program Compared with an Education Program for Survivors of Critical Illness and Their Family Members. A Randomized Clinical Trial. *American journal of respiratory and critical care medicine.* 2018;197(1):66-78.

5. Cox CE, Hough CL, Carson SS, et al. Can Coping-Skills Training Help Patients Who Have Received Intensive Hospital Care to Cope with Depression and Anxiety? 2018.

6. de Miranda S, Pochard F, Chaize M, et al. Postintensive care unit psychological burden in patients with chronic obstructive pulmonary disease and informal caregivers: A multicenter study. *Critical care medicine.* 2011;39(1):112-118.

7. de Ridder C, Zegers M, Jagernath D, Brunnekreef G, van den Boogaard M. Psychological Symptoms in Relatives of Critically Ill Patients: A Longitudinal Cohort Study. *Critical care explorations.* 2021;3(7):e0470.

8. Dijkstra BM, Rood PJT, Teerenstra S, et al. Effect of a Standardized Family Participation Program in the ICU: A Multicenter Stepped-Wedge Cluster Randomized Controlled Trial. *Critical care medicine.* 2024;52(3):420-431.

9. Garrouste-Orgeas M, Max A, Lerin T, et al. Impact of Proactive Nurse Participation in ICU Family Conferences: A Mixed-Method Study. *Critical care medicine.* 2016;44(6):1116-1128.

10. Garrouste-Orgeas M, Flahault C, Vinatier I, et al. Effect of an ICU Diary on Posttraumatic Stress Disorder Symptoms Among Patients Receiving Mechanical Ventilation: A Randomized Clinical Trial. *JAMA.* 2019;322(3):229-239.

11. Gonzalez-Martin S, Becerro-de-Bengoa-Vallejo R, Rodriguez-Garcia M, et al. Influence on Depression, Anxiety, and Satisfaction of the Relatives' Visit to Intensive Care Units prior to Hospital Admission for Elective Cardiac Surgery: A Randomized Clinical Trial. *International journal of clinical practice.* 2022;2022:1746782.

12. Greenleaf B, Foy A, Van Scoy L. Relationships Between Personality Traits and Perceived Stress in Surrogate Decision-Makers of Intensive Care Unit Patients. *The American journal of hospice & palliative care.* 2024;41(6):664-672.

13. Komachi MH, Kamibeppu K. Posttraumatic stress symptoms in families of cancer patients admitted to the intensive care unit: a longitudinal study. *Journal of intensive care.* 2016;4:47.

14. McAdam JL, Fontaine DK, White DB, Dracup KA, Puntillo KA. Psychological symptoms of family members of high-risk intensive care unit patients. *American journal of critical care : an official publication, American Association of Critical-Care Nurses.* 2012;21(6):386-394.

15. Petrinec AB, Mazanec PM, Burant CJ, Hoffer A, Daly BJ. Coping Strategies and Posttraumatic Stress Symptoms in Post-ICU Family Decision Makers. *Critical care medicine.* 2015;43(6):1205-1212.

16. Zante B, Erne K, Grossenbacher J, Camenisch SA, Schefold JC, Jeitziner M-M. Symptoms of post-traumatic stress disorder (PTSD) in next of kin during suspension of ICU visits during the COVID-19 pandemic: a prospective observational study. *BMC psychiatry.* 2021;21(1):477.

17. Amass TH, Villa G, Omahony S, et al. Family Care Rituals in the ICU to Reduce Symptoms of Post-Traumatic Stress Disorder in Family Members-A Multicenter, Multinational, Before-and-After Intervention Trial. *Critical care medicine.* 2020;48(2):176-184.

18. Amass T, Villa G, McFadden R, et al. Family Care Rituals in the Intensive Care Unit to Reduce Symptoms of Posttraumatic Stress Disorder in Family Members-A Multicenter Before-and-After Intervention Trial. *Am. J. Respir. Crit. Care Med.* 2018;197:2.

19. Amass T, Van Scoy LJ, Hua M, et al. Stress-Related Disorders of Family Members of Patients Admitted to the Intensive Care Unit With COVID-19. *JAMA internal medicine.* 2022;182(6):624-633.

20. Azoulay E, Kouatchet A, Jaber S, et al. Noninvasive mechanical ventilation in patients having declined tracheal intubation. *Intensive care medicine.* 2013;39(2):292-301.

21. Bannon S, Lester EG, Gates MV, et al. Recovering together: building resiliency in dyads of stroke patients and their caregivers at risk for chronic emotional distress; a feasibility study. *Pilot and feasibility studies.* 2020;6:75.

22. Beesley SJ, Hopkins RO, Holt-Lunstad J, et al. Acute Physiologic Stress and Subsequent Anxiety Among Family Members of ICU Patients. *Critical care medicine.* 2018;46(2):229-235.

23. Beesley SJ, Hirshberg EL, Wilson EL, et al. Depression and Change in Caregiver Burden Among Family Members of Intensive Care Unit Survivors. *American journal of critical care : an official publication, American Association of Critical-Care Nurses.* 2020;29(5):350-357.

24. Harris BR, Beesley SJ, Hopkins RO, et al. Heart rate variability and subsequent psychological distress among family members of intensive care unit patients. *The Journal of international medical research.* 2021;49(11):3000605211057829.

25. Bohart S, Egerod I, Bestle MH, Overgaard D, Christensen DF, Jensen JF. Recovery programme for ICU survivors has no effect on relatives' quality of life: Secondary analysis of the RAPIT-study. *Intensive & critical care nursing.* 2018;47:39-45.

26. Fumis RRL, Ranzani OT, Martins PS, Schettino G. Emotional disorders in pairs of patients and their family members during and after ICU stay. *PloS one.* 2015;10(1):e0115332.

27. Fumis RRL, Ferraz AB, de Castro I, Barros de Oliveira HS, Moock M, Junior JMV. Mental health and quality of life outcomes in family members of patients with chronic critical illness admitted to the intensive care units of two Brazilian hospitals serving the extremes of the socioeconomic spectrum. *PloS one.* 2019;14(9):e0221218.

28. Garrouste-Orgeas M, Coquet I, Perier A, et al. Impact of an intensive care unit diary on psychological distress in patients and relatives*. *Critical care medicine.* 2012;40(7):2033-2040.

29. Harlan EA, Miller J, Costa DK, et al. Emotional Experiences and Coping Strategies of Family Members of Critically Ill Patients. *Chest.* 2020;158(4):1464-1472.

30. Heesakkers H, van der Hoeven JG, Corsten S, et al. Mental health symptoms in family members of COVID-19 ICU survivors 3 and 12 months after ICU admission: a multicentre prospective cohort study. *Intensive care medicine.* 2022;48(3):322-331.

31. Henderson P, Quasim T, Asher A, et al. Post-intensive care syndrome following cardiothoracic critical care: Feasibility of a complex intervention. *Journal of rehabilitation medicine.* 2021;53(6):jrm00206.

32. Hickman RL, Pignatiello GA, Tahir S. Evaluation of the Decisional Fatigue Scale Among Surrogate Decision Makers of the Critically Ill. *Western Journal of Nursing Research.* 2018;40(2):191-208.

33. Kentish-Barnes N, Azoulay E, Reignier J, et al. A randomised controlled trial of a nurse facilitator to promote communication for family members of critically ill patients. *Intensive care medicine.* 2024.

34. Lester EG, Silverman IH, Gates MV, Lin A, Vranceanu A-M. Associations Between Gender, Resiliency Factors, and Anxiety in Neuro-ICU Caregivers: a Prospective Study. *International journal of behavioral medicine.* 2020;27(6):677-686.

35. Lobato CT, Camoes J, Carvalho D, et al. Risk factors associated with post-intensive care syndrome in family members (PICS-F): A prospective observational study. *J Intensive Care Soc.* 2023;24(3):247-257.

36. Matt B, Schwarzkopf D, Reinhart K, Konig C, Hartog CS. Relatives' perception of stressors and psychological outcomes - Results from a survey study. *Journal of critical care.* 2017;39:172-177.

37. Meyers EE, Presciutti A, Shaffer KM, et al. The Impact of Resilience Factors and Anxiety During Hospital Admission on Longitudinal Anxiety Among Dyads of Neurocritical Care Patients Without Major Cognitive Impairment and Their Family Caregivers. *Neurocritical care.* 2020;33(2):468-478.

38. Meyers EE, Shaffer KM, Gates M, Lin A, Rosand J, Vranceanu A-M. Baseline Resilience and Posttraumatic Symptoms in Dyads of Neurocritical Patients and Their Informal Caregivers: A Prospective Dyadic Analysis. *Psychosomatics.* 2020;61(2):135-144.

39. Meyers E, Lin A, Lester E, Shaffer K, Rosand J, Vranceanu A-M. Baseline resilience and depression symptoms predict trajectory of depression in dyads of patients and their informal caregivers following discharge from the Neuro-ICU. *General hospital psychiatry.* 2020;62:87-92.

40. Milton A, Schandl A, Larsson I-M, et al. Caregiver burden and emotional wellbeing in informal caregivers to ICU survivors-A prospective cohort study. *Acta anaesthesiologica Scandinavica.* 2022;66(1):94-102.

41. Oliveira HSBd, Fumis RRL. Sex and spouse conditions influence symptoms of anxiety, depression, and posttraumatic stress disorder in both patients admitted to intensive care units and their spouses. *Influencia do sexo e condicao de conjuge nos sintomas de ansiedade, depressao e transtorno de estresse pos-traumatico em pacientes admitidos a unidade de terapia intensiva e em seus respectivos conjuges.* 2018;30(1):35-41.

42. Petrinec A. Post-Intensive Care Syndrome in Family Decision Makers of Long-term Acute Care Hospital Patients. *American journal of critical care : an official publication, American Association of Critical-Care Nurses.* 2017;26(5):416-422.

43. Petrinec AB, Martin BR. Post-intensive care syndrome symptoms and health-related quality of life in family decision-makers of critically ill patients. *Palliative & supportive care.* 2018;16(6):719-724.

44. Petrinec A, Wilk C, Hughes JW, Zullo MD, Chen Y-J, Palmieri PA. Delivering Cognitive Behavioral Therapy for Post-Intensive Care Syndrome-Family via a Mobile Health App. *American journal of critical care : an official publication, American Association of Critical-Care Nurses.* 2021;30(6):451-458.

45. Petrinec AB, Wilk C, Hughes JW, Zullo MD, George RL. Self-Care Mental Health App Intervention for Post-Intensive Care Syndrome-Family: A Randomized Pilot Study. *American journal of critical care : an official publication, American Association of Critical-Care Nurses.* 2023;32(6):440-448.

46. Torres J, Carvalho D, Molinos E, et al. The impact of the patient post-intensive care syndrome components upon caregiver burden. *Medicina intensiva.* 2017;41(8):454-460.

47. van Veenendaal N, van der Meulen IC, Onrust M, Paans W, Dieperink W, van der Voort PHJ. Six-Month Outcomes in COVID-19 ICU Patients and Their Family Members: A Prospective Cohort Study. *Healthcare (Basel, Switzerland).* 2021;9(7).

48. Veislinger-Burelli G, Vincent A, Mallard J, et al. Impact of a Visual Support Dedicated to Prognosis on Symptoms of Stress of ICU Family Members: A Before-and-After Implementation Study. *Critical care explorations.* 2021;3(7):e0483.

49. Viana DDR, Santana LB, Azzolin KO, et al. QUALITY OF LIFE AND SATISFACTION OF RELATIVES OF PATIENTS ADMITTED TO INTENSIVE CARE UNITS. *Cogitare Enferm.* 2023;28.

50. Vranceanu A-M, Bannon S, Mace R, et al. Feasibility and Efficacy of a Resiliency Intervention for the Prevention of Chronic Emotional Distress Among Survivor-Caregiver Dyads Admitted to the Neuroscience Intensive Care Unit: A Randomized Clinical Trial. *JAMA network open.* 2020;3(10):e2020807.

51. Wiertz CMH, Hemmen B, Sep SJS, Verbunt JA. Caregiver burden and impact on COVID-19 patient participation and quality of life one year after ICU discharge - A prospective cohort study. *Patient education and counseling.* 2024;123:108221.

52. Cinotti R, Chopin A, Moyer JD, et al. Anxiety and depression symptoms in relatives of moderate-to-severe traumatic brain injury survivors - A multicentre cohort. *Anaesthesia, critical care & pain medicine.* 2023;42(5):101232.

53. White DB, Angus DC, Shields A-M, et al. A Randomized Trial of a Family-Support Intervention in Intensive Care Units. *The New England journal of medicine.* 2018;378(25):2365-2375.
